# Modeling Wildfire Effects on Ecosystem Services in two Disparate California Watersheds and Communities

**DOI:** 10.1007/s00267-025-02185-3

**Published:** 2025-05-23

**Authors:** Ibrahim Busari, Matthew R. Sloggy, Mani Rouhi Rad, Debabrata Sahoo, Stacy A. Drury, Francisco J. Escobedo

**Affiliations:** 1 Clemson University, Agricultural Sciences Department, Clemson, SC USA; 2 USDA Forest Service, Pacific Southwest Research Station, Riverside, CA USA; 3 Texas A&M University, Department of Agricultural Economics, College Station, TX USA

**Keywords:** Wildfire impacts, Regulating ecosystem services, Soil carbon, Socio-demographics, InVEST model, Tubbs Fire, Thomas Fire

## Abstract

Ecosystem services are important for human well-being and for sustaining environmental quality objectives. Growing concern over extreme wildfire events in various watersheds necessitates understanding their impacts on regulating ecosystems services. Past studies have documented how wildfires regulate ecosystem services, but the distributional impacts of such ecosystem services across various human settlements (i.e. communities) remains understudied, despite renewed focus on how they are increasingly at risk from and being impacted by wildfires. We used the Integrated Valuation of Ecosystem Services and Trade-offs (InVEST) model to examine how two wildfires that occurred in California, USA in 2017 impacted water provisioning, soil loss and sediment delivery, carbon sequestration services, and nutrient delivery in two watersheds and their respective communities. Regression analyses were used to determine the differences in the distribution of ecosystem services before and after the fires, and whether these wildfires exacerbated the differences in impacts to ecosystem services across communities in the watershed. We find that a year following the fires, the amount of biomass in forestland, woodland, and chaparral declined in both studied watersheds, while the amount of grassland increased. The model revealed that the changes in vegetation resulted in losing about 200,000 tons of carbon from the Mark West subwatershed and about 160,000 tons of carbon from the southern California watersheds. The expected mean annual water yield for both watersheds increased by 5% and 42%, respectively post-fire. Expected post-fire phosphorus and nitrogen export also increased. Finally, we found evidence of human community-level differences in the distribution of pre-fire ecosystem services but no evidence that post-fire conditions either exacerbated or alleviated these impacts.

## Introduction

Watersheds provide a variety of ecosystem services including carbon sequestration, water quantity and quality improvement, and regulation of pollution levels (Brockerhoff et al. 2017). These ecosystem services are vital for sustaining life and achieving environmental management and quality goals. A major threat to the sustainable supply of ecosystem services is the increased frequency and severity of wildfires due to land use and climate change, in more arid regions of the Western US (Westerling et al. 2006), Australia (Haque et al. 2021), western and southern Europe (Dupuy et al. 2020) and South America (Ciocca et al. 2023). While wildfires are part of many properly functioning ecosystems (Lecina-Diaz et al. 2021), changing wildfire regimes will likely result in changing levels of ecosystem service provision on severely and frequently fire-affected watersheds (Pereira et al. 2021).

**Fig. 1:**
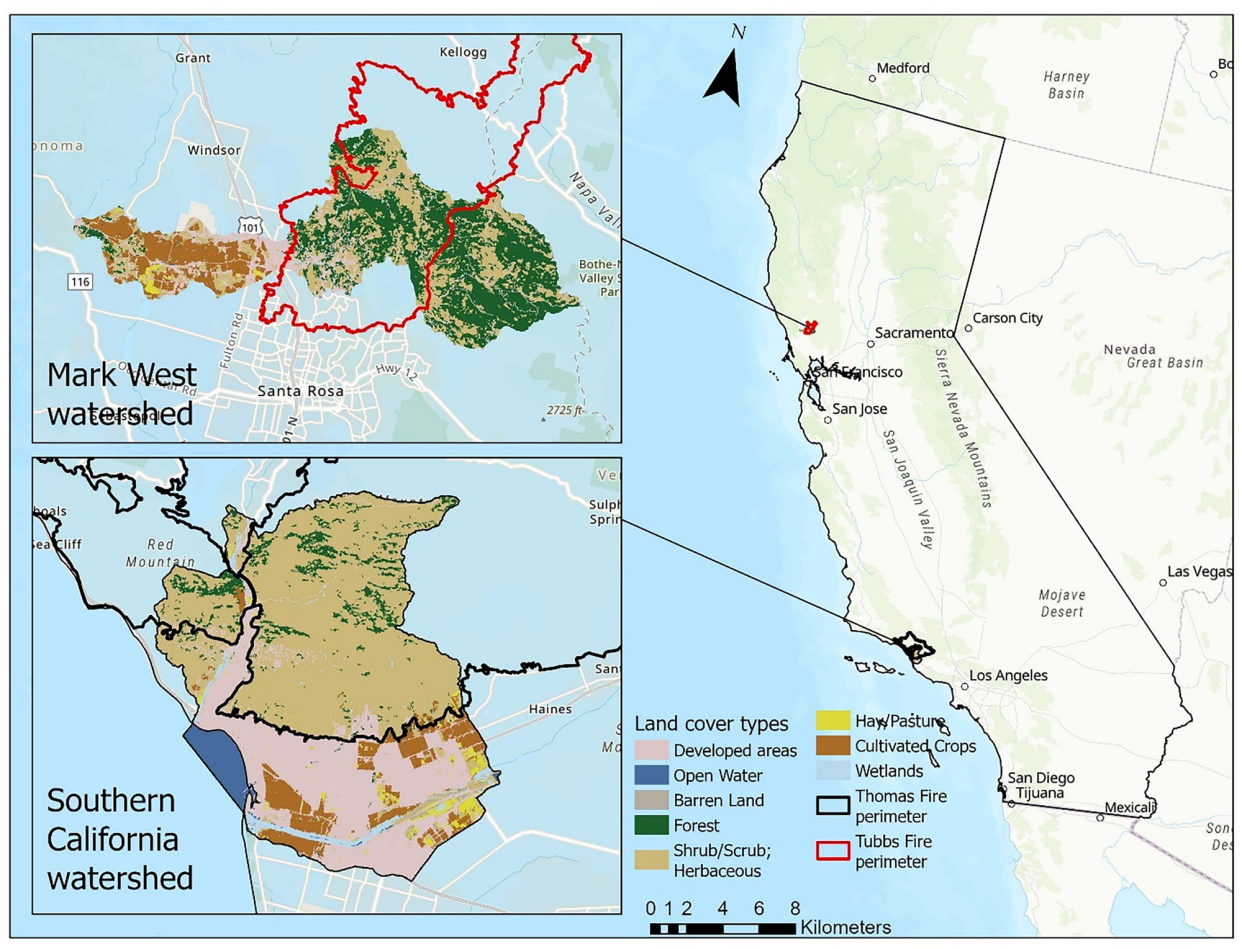
Map of the study area with land cover types

This study focuses on the effects of wildfires on regulating Ecosystem Services (ES) in two watersheds along with the change in the distributional impacts of ecosystem services across local communities. The study area includes two fire-affected landscapes: the Mark West subwatershed before and after the Tubbs fire, and Harmon Canyon, Arundell, and Ventura subwatersheds representing the southern California watersheds in Ventura and Santa Barbara County which were impacted by the Thomas Fire (Fig. 1). The diversity in watersheds provided by these two areas makes them useful for this topic, with the northern California landscape consisting of oak savanna and riparian woodlands, and the southern California landscape consisting of chaparral. In addition, California has experienced an increase in the frequency and severity of wildfires throughout the state partly due to climate change as well as human-driven factors (Westerling and Bryant 2008). With increasing climate and land use changes, the state is experiencing increases in wildfire risk (Westerling and Bryant 2008). This makes it an ideal and important area to consider when studying how fires might impact the equitable and sustainable distributions of ecosystem services.

Wildfires can have a range of negative effects on ecosystems. Post wildfire events such as heavy storms, ash and debris runoff, and vegetation loss may lead to increased soil loss and sediment export including water quality degradation, reduction of nutrient-enriched soil, habitat destruction, reduced soil productivity, and water scarcity (Lopes et al. 2021). Carbon losses within forest ecosystems may also be attributed to wildfires due to burning of plant biomass and organic soils layers, although the extent of this effect vary with tree species, fire regime, slope, elevation and the time since the fire (Cheng et al. 2023). In addition, soil loss associated with wildfires might compromise water quality by increasing turbidity and introducing pollutants, which could contaminate downstream rivers, lakes, and reservoirs (Issaka and Ashraf 2017). Soil erosion will lead to the loss of essential topsoil, which is enriched with nutrients, reducing soil fertility, and impacting plant growth and productivity (Orgiazzi and Panagos 2018). Sedimentation of rivers, reservoirs, ponds, and channels is often attributed to increased soil loss from the watershed, and sediment export reduces the water carrying capacity of the waterbody and may cause overflow, leading to flooding (Uri 2001). Soil erosion may also threaten terrestrial biodiversity by impacting communities of the fauna inhabiting the soil through habitat degradation (Guerra et al. 2020),

However, not all the effects of wildfires are deleterious. Due to reduced interception losses and higher net precipitation, wildfires have also result in increased water yield and peak flows (Maina andSiirila-Woodburn 2020). Changes in streamflow regime are also associated with wildfire events, which may have substantial impacts on downstream aquatic ecosystems (Wampler et al. 2023). Elevated base flows help maintain cool stream temperatures during critical summer months and also provide additional water supplies to downstream communities (McGuire et al. 2017). Furthermore, fire-restored landscapes can lead to increased water yield due to restoration of springs and seeps (Boisramé et al. 2017, 2019; Stevens et al. 2020). Finally, soil erosion can lead to greater diversity of benthic habitat as downstream waterbodies that are sediment poor.

In addition, increased nutrients such as nitrogen and phosphorus in streams have been associated with post wildfire events in temperate, Mediterranean and boreal forests (Rust et al. 2018; Emelko et al. 2016; Sherson et al. 2015). Nutrient responses in streams vary with post fire storm intensity, with rapid response observed in storms immediately after fires (Harris et al. 2015), while delayed responses are reported in arid environments (Murphy et al. 2015). Nutrient concentrations may remain elevated but typically revert to reference norms within a median of 5 years, with phosphorus levels often taking somewhat longer than nitrogen to restore to pre-fire conditions on average (Paul et al. 2022).

We contribute to the literature on the effects of wildfires in two ways. First, several studies have analyzed the effects of fires on ecosystem services provided by forested watersheds. Lecina-Diaz et al. (2021) studied the risk to forest-based ecosystem services based on a hazard index that depends on the amount of at-risk forest ecosystems, the probability of fire hazard from weather, and the capacity of the forest to recover after a fire. Lee et al. (2015) studied the benefits of climate change mitigation by studying their benefits on reducing the loss of ecosystem services from forests. They used a habitat equivalency analysis that estimates the loss of ecosystem services as acre-years of lost vegetation and considered the avoided cost of fuel treatment as a benefit of mitigation. We contribute to this literature by studying the changes in regulating ecosystem services provided by forests across watersheds in two different regions of California.

Second, several studies have examined how ecosystem services are distributed across human settlements (Plieninger et al. 2013; Fu et al. 2015), and many have addressed the effects of wildfire on ecosystem services (e.g., Vukomanovic and Steelman 2019). But, there remains a gap in examining how wildfires change the spatial distribution and supply of ecosystem services across different communities in a watershed (Yadav et al. 2023). Core to this paper is how wildfire may be related to community well-being across different socio-demographic groups (Thomas et al. 2022). In this study, we examine specifically the distribution of ecosystem services, which are defined as benefits received by individuals that flow from environmental sources (Chen et al. 2023). The ecosystem services are distributed spatially across the landscape, which influences how they benefit different sociodemographic groups. In addition to ecosystem services, the landscape can also produce ecosystem disservices, which are defined as costs incurred by individuals (Escobedo et al. 2011). Wildfire changes the spatial distribution of the ecosystem services, and thus changes the distributional impacts of these as well. In addition to ecosystem services and disservices being distributed across the physical landscape, they can also be distributed across different communities living in and around the watershed. To address this, once we map ecosystem services and disservices across the physical landscape, we link it to spatially explicit sociodemographic data on communities.

The aim of this paper is to model the spatiotemporal and distributional impacts of wildfires on watershed-scale ecosystem services across different communities. The specific objective of this study is to (1) model the effects of two wildfires in different ecoregions and watersheds in California had on: i. water quantity, ii. soil loss and sediment delivery, iii. carbon sequestration, iv. nutrient delivery and (2) understand the pre- and post-fire spatial and distributional impacts to ES supply across different sociodemographic groups in two communities. The main contribution of this study is the novel linkage of fire and ecosystem service modeling to sociodemographic analysis to estimate the distributional impacts of wildfires on ecosystem services.

## Methodology

### Study Area

Our study focuses on the Mark West subwatershed in northern California, and the Harmon, Arundel and Ventura subwatersheds in southern California hereafter called Southern California watershed. These two watersheds were chosen because they were subject to large wildfires which greatly impacted communities while at the same time are located in two separate ecoregions. The differences in size, climate, land cover, and other biophysical attributes between these two watersheds can be used to better understand the heterogeneity in the effects of wildfires on ecosystem services.

The Mark West subwatershed is considerably smaller (14,767 hectares) than the southern California watershed (26,379 hectares) (Fig. 1 right panel). Further, the subwatershed is at an altitude of between 5 meters and 850 meters above sea level (Woolfenden et al. 2011). The watershed of the Mark West Creek has a mediterranean climate and is part of a larger region that is characterized by several features including oak woodlands, grasslands, and riparian woodlands (Potter and Hiatt 2009).The southern California watershed is coastal and has a maximum altitude of 1833 m at its headwaters and has a Mediterranean climate and characterized by large amounts of chaparral vegetation (Jumps et al. 2022). A map of the vegetation cover is presented in Fig. S1 of the supplementary document.

The Mark West subwatershed was burned during the 2017 Tubbs fire. The Tubbs fire burned through parts of Sonoma County, affecting many populated areas including the community of Santa Rosa, Sonoma county’s largest city (Cortenbach et al. 2019). The fire destroyed over 5643 structures and 22 people lost their lives (LeComte 2018). The southern California watershed also burned in 2017 during the Thomas fire. The fire burned through and affected communities in Ventura and Santa Barbara counties (Kolden and Henson 2019) and resulted in a large landslide that destroyed parts of Highway 101 south of Santa Barbara (Lukashov et al. 2019). The fire and mudslide that followed resulted in 23 people being killed (Kress 2020). More detailed accounts and descriptions of both fires including history, severity and return interval can be found in supplementary document. Additional details regarding the fire can be found in the supplementary information.

### Fire Effects Modeling Process

The fire effects modeling process was designed to provide pre- and post-burn above ground biomass data for the 2017 Tubbs fire in northern California and the 2017 Thomas Fire in southern California. Both fires started outside the Wildland-Urban Interface (WUI) and burned into the urban communities of Santa Rosa CA and Ventura CA. For this process, fire perimeter data is downloaded from the monitoring trends in burn severity project (MTBS; Eidenshink et al. 2007). Pre-burn above ground biomass is estimated using the fuel characteristic classification system maps (Fuel Characteristic Classification System; Ottmar et al. 2007, Prichard et al. 2013) as provided by the LANDFIRE project data distribution site (Rollins 2009). Initial fire severity observations are downloaded from the Rapid Assessment of Vegetation Condition (RAVG; less than 1-month post-fire; Miller and Thode 2007; Miller and Quayle 2015) followed by downloading 1-year post-fire observations from MTBS (Eidenshink et al. 2007). Post-burn above ground biomass is estimated based on biomass reduction equations using the fire severity observations (Prichard et al. 2017) and the fire and fuels tools software package (Prichard et al. 2013).

Specifically, we used the fire perimeters created by MTBS to define the area affected by fire. The MTBS project uses Landsat earth observations taken of the general fire area before the fire and then after the fire in combination with fire perimeters gathered by GIS specialists to determine the fire perimeter which becomes the final perimeter of record. The study area was further defined to the areas covered by the Mark West Creek watershed for the Tubbs Fire and the Lower Ventura River, Arundell Barranca-Frontal Pacific, and the Harmon Canyon-Santa Clara River watersheds for the Thomas Fire from the California HUC12 watershed delineation maps. Each of these watersheds contained significant portions of urban and WUI landscapes in the watershed that were burned by wildfire.

Pre-fire vegetation type was estimated for each 30 square meter pixel within the watershed boundaries using the Fuel Characteristic Classification System (FCCS) layer included in the 2016 release of the LANDFIRE fuels and vegetation layers (www.landfire.gov) and the LANDFIRE existing vegetation layer (EVT: Rollins 2009). The LANDFIRE EVT and the LANDFIRE FCCS layers each contain vegetation type descriptions that progress from generic coarse scale vegetation type descriptions such as “shrubland”, “conifer” or “hardwood” to fine scaled descriptive names including “California Coastal Live Oak Woodland and Savanna”. To simplify the analysis, we used the more generic coarse scale descriptions since few pixels in our landscapes contained the more specific vegetation types. Above ground biomass was estimated using the LANDFIRE FCCS layer and associated database (Pritchard et al. 2017). FCCS provides biomass estimates in biomass per unit area such as tons per acre (Ottmar et al. 2007), and can be summed into the following broad categories: tree, shrub, herbaceous, downed and dead logs, and forest floor biomass (new and decomposed biomass). We used ArcMap 10.5 and excel spreadsheets to link the EVT maps with the FCCS maps to produce the pre-burn estimates by vegetation type for each of our two large watersheds. We examined the impacts that the Tubbs and Thomas fires had on the landcover distributions of the Mark West and Southern California Watersheds.

Biomass changes across the fire affected landscapes in the watersheds were estimated using fire severity metrics provided by MTBS (Eidenshink et al. 2007). Fire severity is estimated by quantifying vegetation reflectance differences where Landsat imagery is compared before and after fire using the relative differenced Normalized Burn Ratio (Eidenshink et al. 2007). The quantified difference is then related back to the original vegetation to estimate changes in vegetation condition, status (live or dead), and biomass consumed (Drury et al. 2014; Prichard et al. 2017).

This methodology does not enable us to determine changes in vegetation type but does provide tools to estimate biomass remaining on the landscape after burning (Drury et al. 2014; Prichard et al. 2017). However, we estimated the potential impact of the modeled wildfire on land cover by aggregating the number of cells in each landscape before and after the fire and estimating the percent change (Table S1). Specifically, we combined the fire severity maps with post-fire biomass remaining calculations produced by the FCCS Fire and Fuels Tools (Ottmar et al. 2009) and post-fire biomass equations developed for the LANDFIRE mapping project (Prichard et al. 2017) using ArcGIS 10.5 to produce a custom set of biomasses remaining in the fire affected areas of the Tubbs and Thomas fire perimeters. The resulting pre- and post-burn biomass maps serve as inputs into the InVEST model described below.

### Ecosystem Services Modeling

To achieve our first objective, we used four different InVEST modules to model five different ecosystem services that are affected by wildfires: (1) carbon storage, (2) carbon sequestration, (3) annual water yield, (4) sediment delivery, and (5) nutrient delivery (phosphorus and nitrogen). The InVEST model has previously simulated the effect of climate and land use-cover change on ecosystem services like water yield and supply (Clerici et al. 2019; Fu et al. 2017). Changes in each ecosystem service in this study were estimated by comparing modeled ecosystem services before and after fire for each watershed. Land cover alterations that occur after the fire event describe the impact of the fire in each watershed on existing vegetation cover. We first estimated the outcome of each ecosystem process using the vegetation cover prior to the fire for each watershed. We then estimated the ecosystem outcomes for the vegetation cover one year after the fire. The difference between the two provided us with the change in ecosystem services as a result of the fires. The reason that we selected one year after each fire as our post-fire ecosystem service assessment is that vegetation changes are more stable a year after a fire as opposed to modeling day-to-day changes in vegetation and ecosystem services immediately after a fire, which may not provide an accurate representation of ecosystem service changes. We calculated the differences between the InVEST model outputs prior to and following the fire by loading both outputs as rasters into R (R Core Team 2022) and using the *raster* package (Hijmans 2023) to take the difference in values between the two rasters. Since the InVEST modules requires a set of different inputs so we parameterized each module using a variety of inputs listed in Table 1.

**Table 1:** InVEST Input data details

Input	Type	Sources	Units
Rainfall Erosivity index (R)	Raster	Global Rainfall Erosivity Database (Panagos et al. 2017)	MJ.mm.(ha.h.yr)^−1^
Soil Erodibility (K)	Raster	gSSURGO (Soil Survey Staff 2020)	t.ha.hr.(MJ.mm.ha)^−1^
Digital Elevation Model (m)	Raster	SRTM (Farr et al. 2007)	m
Precipitation	Raster	Daymet (Thornton et al. 2022)	mm
Support practice factor (P)	Decimal	InVEST User guide (Sharp et al. 2020)	Unitless
Cover management factor (C)	Decimal	Literature (Tetra Tech 2015b; McKague 2023; Terranova et al. 2009)	Unitless
Crop coefficient	Decimal	Literature (Nistor et al. 2018)	Unitless
Plant Available Water	Raster	gSSURGO (Soil Survey Staff 2020)	mm
Reference Evapotranspiration	Raster	Daymet (Thornton et al. 2022)	mm
Depth To Root Restricting Layer	Raster	gSSURGO (Soil Survey Staff 2020)	mm
Borselli k Parameter	Decimal	Vigiak et al. (2012)	Unitless
Borselli IC0 Parameter	Decimal	Vigiak et al. (2012)	Unitless
Threshold flow accumulation	Integer	Vigiak et al. (2012)	Unitless
Max SDR Value	Decimal	Vigiak et al. (2012)	Unitless

#### Annual Water Yield

The annual water yield module of InVEST quantifies the contribution of different parts of the watershed to the overall water reaching the outlet in a year (Wu et al. 2022). This refers to all forms of water movement that originate from precipitation, snowmelt, and other sources in the watershed. The annual water yield of a watershed is an essential ecosystem service that supports human life and development. The InVEST annual water yield module estimates the water yield for a watershed at the pixel-level and at the watershed-level water (Sharp et al. 2020). This module can also estimate the economic value of energy produced using the water supplied to the hydropower reservoir based on the contributions of water runoff from each landscape type (Sharp et al. 2020). Hydropower is a vital energy source in California (Tarroja et al. 2019); however, extending our analysis to include hydropower was deemed out of scope. Generally, the model determines the quantity of water yield per pixel as the difference between precipitation and evapotranspiration (ET). In this study, we estimate the change in the annual water yield of each watershed due to burning by using the InVEST annual water yield module to first estimate water runoff from each pixel and aggregate runoff for each watershed before and after the relevant fire event. Then, the difference between annual yield before and after a fire is presented as the effect of wildfires on the change in annual water yield.

Estimating annual water yield using the module requires input data on precipitation, biophysical information, evapotranspiration, plant available water content, root restricting layer depth, and consumptive water use. Precipitation data was obtained from Daymet (Thornton et al. 2022) for the period of 1987–2017 based on the tile numbers that represent the study area. Daymet is a data-driven product that uses various algorithms for interpolation and extrapolation of daily meteorological parameters to produce gridded daily parameters at a spatial resolution of 1 km. Daily precipitation values in millimeters were summed by year and then cropped to the study watershed areas to obtain the average annual precipitation estimates. The minimum and maximum temperature and solar radiation were also downloaded from the same database and used for the estimation of the reference evapotranspiration (ETo) based on the modified Hargreaves equation. These meteorological variables used as model input are historical average across the 30 years of data acquisition. Data on Depth to Root Restricting Layer was obtained from the Gridded Soil Survey Geographic (gSSURGO) database (Soil Survey Staff 2020). Similarly, Plant Available Water Content Fraction which is the ratio of actual ET and precipitation was obtained from the Gridded Soil Survey (gSSURGO) database. Watershed shapefiles were secured from the U.S. Geological Survey’s Watershed Boundary dataset while the same land use /land cover details (as in the case of the carbon model) were applied to the annual water yield model estimation.

Additional data efforts focused on creating biophysical parameters and water demand information relevant to the water yield model estimation. Accumulated biophysical data includes land use/land cover codes for each landscape class, crop coefficients (Kc values), root depth, and Z-parameter. Land use/land cover codes are integers and remain the same as those used in the carbon model. Kc values for each land cover classification were secured from Nistor et al. (2018). Root depth for each land cover type was also obtained from published studies (Canadell et al. 1996). Maximum root depth for each vegetation type measures the depth to which at least 95 percent of root biomass occurs. Finally, we calculated the Z-parameter using omega estimates based on the work of Xu et al. (2013), and the mean of precipitation and available water content earlier described. We focused on the Mark West subwatershed and the southern California watershed, estimating water yield before and after fire events.

#### Sediment Delivery

By altering vegetation cover and litter, fires can also change the amount of sediment exported from a catchment (Warrick et al. 2012). The sediment delivery ratio module of the InVEST model was used to assess the annual sediment exported from the catchment to the outlet. The model uses a combination of the soil loss calculated through the revised universal soil loss equation (RUSLE) and the sediment delivery ratio (SDR), which quantifies the proportion of soil loss reaching the outlet. The model works explicitly on the spatial resolution of the digital elevation model (DEM) and performs its operation for each pixel (Sharp et al. 2020). The SDR estimation begins by computing the hydrological linkage between sediment sources and streams, often called connectivity index (IC), which is a function of the area upslope of each pixel and the flow path between the pixel and the nearest stream.

The data sources for the InVEST SDR model ranges from literature, organizations, public reports and agencies. The rainfall erosivity index, (R hereafter), quantifies the intensity of rainfall to initiate soil loss and was obtained from the global erosivity map published by the Joint Research Center of the European Commission. The map was a result of an extensive project focused on estimating rainfall erosivity across 63 countries (Panagos et al. 2017). The soil erodibility factor (K) was derived from the United States Department of Agriculture’s NRCS gSSURGO database and measures the ability of the soil to be eroded under standard condition. The R and K factor for the study sites were clipped out of the raster map obtained from their respective databases. The Digital Elevation Model (DEM) raster file was obtained from the Shuttle Radar Topography Mission (SRTM) database and processed using ArcGIS Pro 3.0.3. The Fill Sink tool in the software was used to fill the depressions in the DEM. The threshold flow accumulation and Borselli K parameter were set at 1000 and 2, respectively. The cover management factor (C), utilized in this study quantifies the ability of a land use type to resist erosion. This value ranges from 0 to 1, with values closer to 0 indicating that less erosion is likely to occur while values closer to 1 indicate more is likely to occur in the land use pixel. The C-factor for the pre-fire land cover map for the watersheds was obtained from ensemble sources including literature, sediment database provided in the InVEST User guide, and technical reports such as Tetra Tech (2015b) and McKague (2023). The C-factor for post-fire land cover map was however derived based on results from published literature (Terranova et al. 2009), which assigns a C-factor to land cover based on burn severity. This study assigns C = 0.20 for severely burned areas, C = 0.05 for moderately burned areas, and C = 0.01 for areas that had burned at low severity. The support practice factor (P) was set to 1 since no land management practice was identified in the study site (Sharp et al. 2020). The maximum theoretical SDR was set as 0.8 as recommended by Sharp et al. (2020), and the K and *IC*_0_ parameters are set to 2 and 0.5 respectively as explained by Vigiak et al. (2012).

#### Carbon Storage and Sequestration

Carbon sequestration and storage is an important climate regulating ES provided by forests (Sohngen and Brown 2006), and carbon emissions are a notable ecosystem disservice arising from wildfires (Simmonds et al. 2021). To evaluate the impacts of the Tubbs and Thomas fires on the carbon storage within our watersheds of interest, we first estimated changes in the biomass levels arising from wildfire-induced vegetation changes (see Section 3.1). We then compared the aboveground carbon levels across both scenarios. We assume no land use changes before and after each fire. In practice, there could be changes in land, which has been demonstrated in other studies (Mockrin et al. 2020). We convert above ground biomass (AGB) per acre to Carbon (or Carbon equivalent, as opposed to Carbon dioxide equivalent) per hectare using Eq. (1):

1 $${C}_{{hectare}}=2.471* {{AGB}}_{{acre}}=1.2355* {{AGB}}_{{acre}}$$

The factor 2.471 is the conversion from Acres to Hectares, and 0.5 is the factor that converts dry weight biomass to carbon (as opposed to Carbon Dioxide equivalent; Li et al. 2011; Wirasatriya et al. 2022). Once the conversion factor is applied to the biomass stores before and after the fire, we subtracted the total above ground carbon stored before the fire from the total above ground carbon stored after the fire and calculated the change in carbon pre- and post-fire.

#### Nutrient Delivery

The nutrient delivery module of InVEST was used to quantify the export and retention of nitrogen and phosphorus across the watersheds and to identify changes in nutrient export under land cover conditions before and after the fires in the two study areas. The module uses the simple mass balance concept to describe the long steady state flow of nutrients using empirical relationships (Sharp et al. 2020). The model computes the nutrient export from each pixel based on nutrient sources on each LULC and the retention properties of the pixels belonging to the same flow path (Pärn et al. 2012). The nutrient sources refer to nutrient applications across the LULC in the form of loadings and could be surface and subsurface sources (Hanshaw et al. 2009).

The DEM raster map utilized as input for this model was identical to the one for the SDR model. The LULC maps utilized for the scenario experiment were the pre- and post-fire land cover maps. The nutrient runoff proxy for this study was the annual precipitation downloaded from Daymet. The runoff proxy is used to evaluate the spatial variability of runoff which has the capacity to transport nutrient downstream. The biophysical table for this model was parameterized with data from an extensive literature search. For the pre-fire nutrient delivery modeling, the nitrogen and phosphorus loadings for each unique land cover type were obtained from the nutrient analysis report prepared by Tetra Tech (2015a), Fenn et al. (2010), and nutrient database provided in the InVEST User guide. The retention efficiency (eff) for each nutrient is the maximum nutrient retention expected from each LULC type. This ratio varies from 0 to 1, with high values (0.6–0.8) assigned to natural vegetation, indicating that 60–80% of nutrients are retained by these land cover (Sharp et al. 2020). The critical flow length which describes the distance of travel required to achieve the nutrient retention coefficient was set to the resolution of the input LULC raster map. The proportion subsurface n and Borselli K parameter values were obtained from the user guide of the InVEST NDR module (Sharp et al. 2020). For the post-fire modeling, the nutrient loadings required as input of the NDR module were derived from published literature that focused on the contributions of wildfire on nutrient deposition. Based on the study of Koplitz et al. (2021) and Wright (1976), a 30% increase in Nitrogen loadings and about 38% increase in Phosphorus loadings were used to calibrate the post fire NDR biophysical table. Input data details and sources are provided in Table 1.

### Analysis of Wildfire Impacts to Communities

To test our second objective, we assessed the extent to which the Tubbs and Thomas fires led to changes in how ecosystem services and disservices are distributed across different communities, pre- and post-fire. We used Ordinary Least Squares (OLS) regression (Eq. (2)) to investigate the extent to which several sociodemographic variables are associated with changes in water yield, soil loss, nitrogen loading, and phosphorus loading. Specifically, we overlayed the output rasters from each InVEST module on California Communities Environmental Health Screening Tool’s (CalEnviroScreen) US Census tracts (CEPAO 2017). The spatial overlay allowed us to attribute InVEST grid cells to US Census tracts. Each census tract had various InVEST grid cells attributed to it. To estimate a tract-level quantity for each InVEST variable, we took the mean of grid cells attributed to a particular census tract. As opposed to previous analyses described above, the data for the socio-demographic analysis were pooled together to ensure that the analysis has sufficient statistical power.

2 $${y}_{i,h}={\beta }_{0}+{\beta }_{1}U+{\beta }_{2}P+{\beta }_{3}E+{\beta }_{4}H+{\beta }_{5}L+\epsilon$$

Where ${y}_{i,h}$ is the InVEST variable before the fire (subscript *b*) for a given US Census tract (subscript *i*). The explanatory variables of the regression include the unemployment rate, *U*, the poverty rate *P*, the Education level *E*, housing burden *H*, and the linguistic isolation, *L*. All explanatory variables are included as percentages. The definition of the unemployment rate, per OEHHA (2021), is: “Percent of the population over the age of 16 that is unemployed and eligible for the labor force” and poverty rate is defined as “Percent of population living below two times the federal poverty level”. The education level is defined as “Percent of population over 25 with less than a high school education” (OEHHA 2021). Housing burden is defined as “Percent housing-burdened low-income households” (OEHHA 2021). Finally, linguistic isolation is defined as “Percent limited English speaking households” (OEHHA 2021). The error term is given by e and is assumed to be normally distributed and mean zero. The constant is *β*_0_, with the coefficients of the regression being the various *β*s.

Next, we ran a second OLS regression that examines the relationship of the sociodemographic variables with differences in the ecosystem services simulated by the InVEST before and after the fire, conditioning on the pre-fire levels of the ecosystem services simulated by InVEST.

3 $${y}_{i,a}-{y}_{i,b}={\beta }_{0}+{\beta }_{1}U+{\beta }_{2}P+{\beta }_{3}E+{\beta }_{4}H+{\beta }_{5}L+{\beta }_{6}{y}_{i,b}+\epsilon$$

Where the variables in the above regression are the same as in Eq. (1), with the exception that the dependent variable is the difference before and after the fire ${y}_{i,a}-{y}_{i,b}$ and the InVEST variable before the fire is included as an explanatory variable ${y}_{i,b}$.

The effects of outliers are a larger concern for datasets with fewer observations. To limit the impact of outliers on the regression, we apply a hyperbolic arcsine transformation to all variables in Eqs. (2, 3). An added benefit of the hyperbolic arcsine transformation is that the interpretation of the coefficients in the regressions become approximations of percent changes (Bellemare and Wichman 2020). Thus, the interpretation of any given coefficient from estimating Eqs. (2) or (3) were that a 1 percent change in each sociodemographic variable on average results in a $\beta$ percent change in either the pre-fire level of ecosystem service (Eq. (2)) or the difference in pre- and post-fire ecosystem services, all in a given census tract (Eq. (3)). For all statistical analyses we used the lfe software package (Gaure 2013).

## Results

### Annual Water Yield

**Fig. 2:**
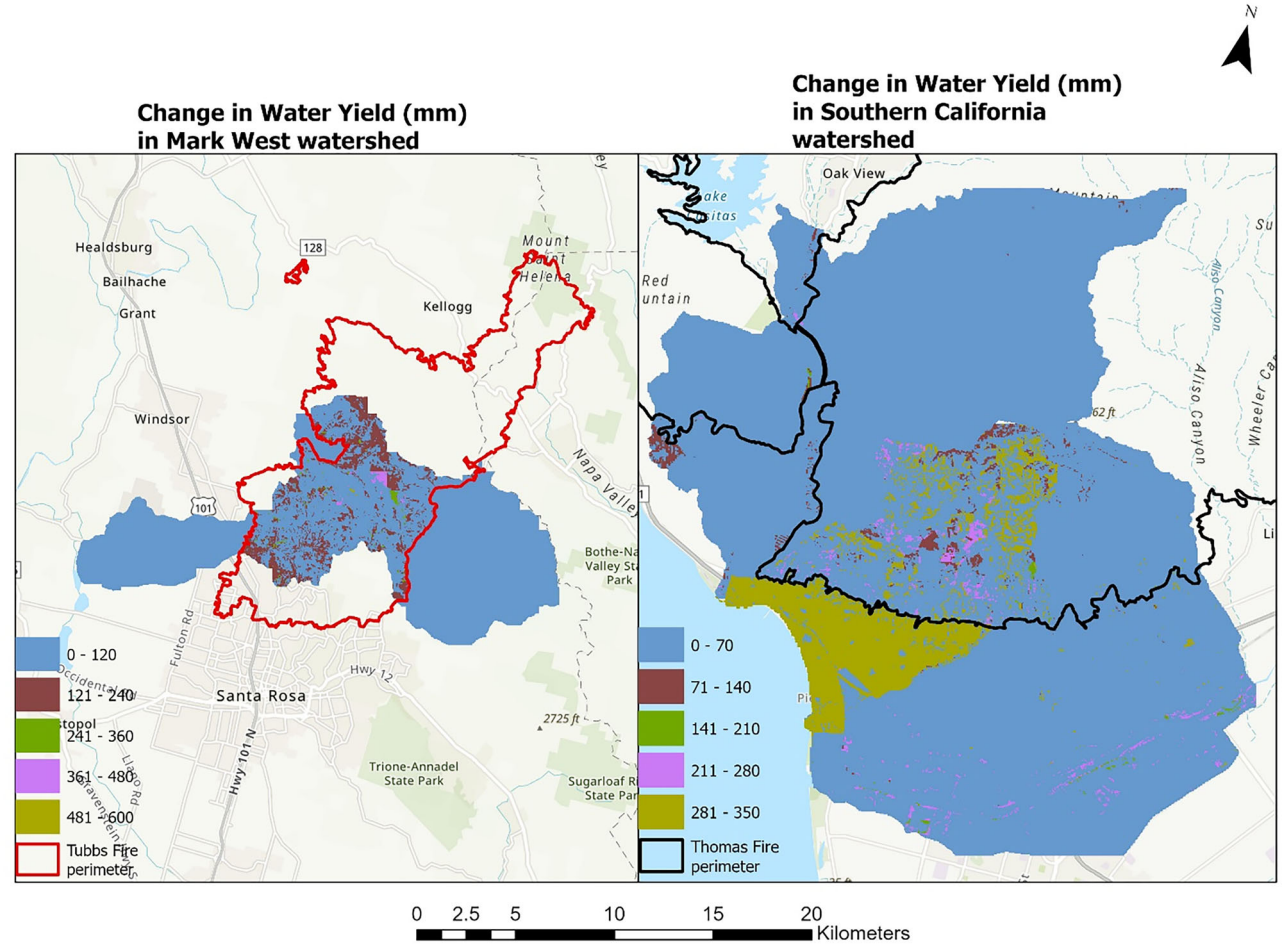
The figure shows the difference in annual water yield (mm) before and after the fire in the Mark West (left) and southern California watersheds (right)

The mean modeled annual water yield for the Pre-fire scenario in the Mark West subwatershed ranged between 168 mm and 945 mm, with a mean value of 699 mm. Post-fire land use map-based simulations show that the modeled mean annual water yield increased by 6% with a spatial range of 168 mm and 1032 mm, while the modeled spatial difference between the pre- and post-fire scenario ranges between 0 and 590 mm as shown in Fig. 2. A decrease in modeled actual evapotranspiration was also observed for the post-fire modeling scenario compared to the pre-fire scenario.

Similarly, an increase in modeled annual water yield was observed in the southern California watersheds after the fire event. The spatial variation in the modeled water yield upon comparing the pre- and post-fire scenario ranges between 0 and 347 mm as shown in Fig. 2. A difference of about 8.4 million m^3^ was obtained in the modeled total annual water yield in the watershed, indicating a 42% increase in the modeled post fire annual water yield compared to the pre-fire scenario. The increase in modeled water yield could be attributed to the decrease in evapotranspiration and reduced interception due to changes in land cover.

### Soil Loss and Sediment Delivery

**Fig. 3:**
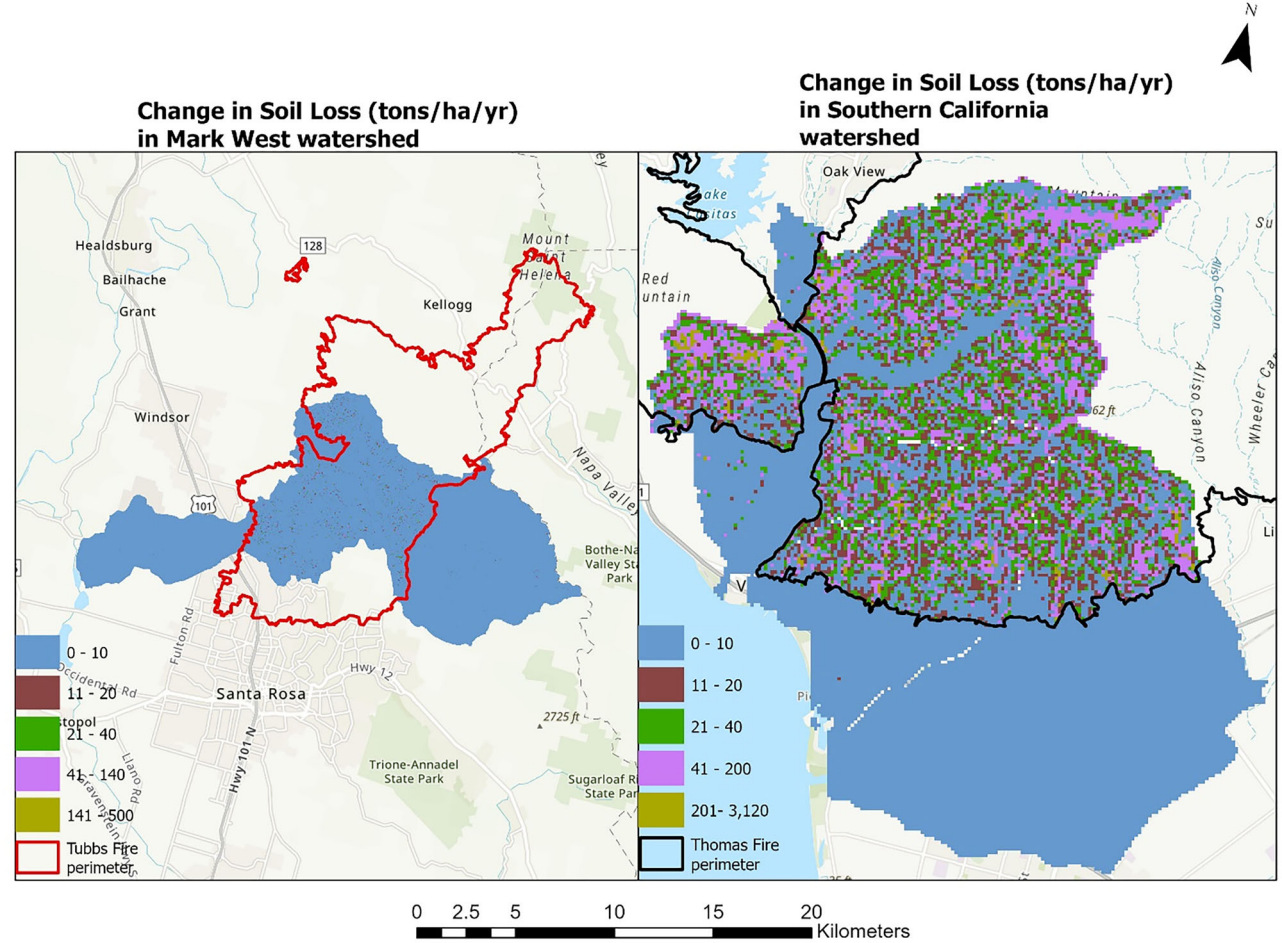
The figure shows the difference in soil loss (tons ha^−1^ year^−1^) before and after the fire in the Mark West (left) and southern California watersheds (right)

The land cover alteration due to the fire affects the soil loss in both watersheds. The modeled total soil loss was about a 66% increase in the Mark West subwatershed (Table 2). An increase was also observed in the southern California sub-watersheds, where the modeled post-fire soil loss increased to 578,779 tons from about 567,335 tons before the fire event as shown in Table 3. The modeled spatial difference between the pre- and post-fire soil loss in the Mark West subwatershed ranges between 0 to 486 tons ha^−1^ year^−1^ and in southern California ranges from 0 to 3,116 tons ha^−1^ year^−1^, as shown in Fig. 3.

**Table 2:** Mark West Sub-watershed ecosystem service modeling

Category	Pre-fire	Post-fire	%Change
**Mark West Annual Water Yield**			
Water Yield (mm^3^/yr)	103,335,478	108,045,478	5
**Mark West Soil loss and Sediment Delivery**			
Soil loss (tons)	541,020	898,726	66
Sediment export (tons)	44,332	94,293	113
Sediment deposition (tons)	377,916	612,338	62
**Mark West Nutrient Delivery**			
Nitrogen loads (Kg)	28,635	31,705	11
Nitrogen export (Kg)	4758	5297	11
Phosphorus loads (Kg)	7652	8785	15
Phosphorus export (Kg)	1248	1447	16

**Table 3:** Southern California watersheds ecosystem service modeling

Category	Pre-fire	Post-fire	%Change
**Southern California Annual Water Yield**			
Water Yield (mm^3^/yr)	19,977,405	28,366,687	42
**Southern California Soil loss and Sediment Delivery**			
Soil loss (tons)	567,335	578,779	2
Sediment Export (tons)	23,118	24,308	5
Sediment deposition (tons)	310,534	285,414	8
**Southern California Nutrient Delivery**			
Nitrogen loads (Kg)	99,219	122,646	24
Nitrogen export (Kg)	13,651	15,586	14
Phosphorus loads (Kg)	35,513	45,910	29
Phosphorus export (Kg)	5058	6690	32

**Fig. 4:**
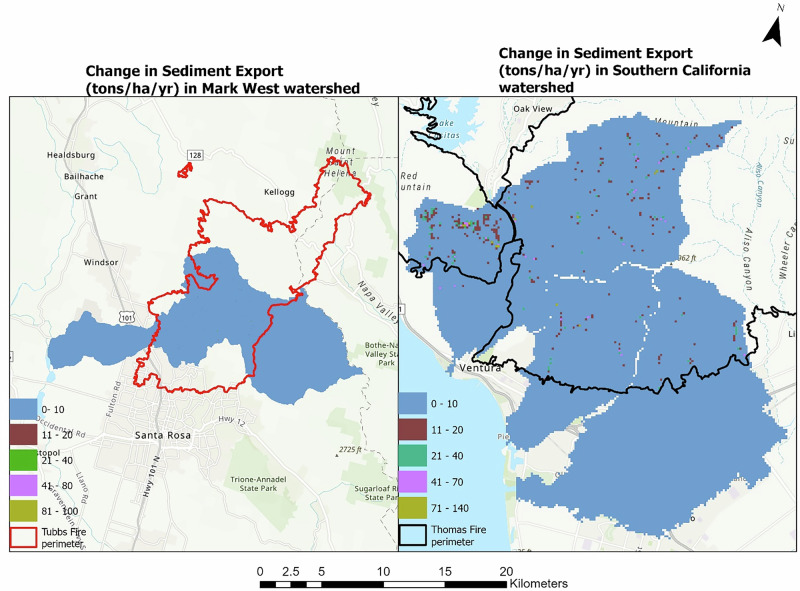
The figure shows the difference in sediment export (tons ha^−1^ year^−1^) before and after the fire in the Mark West (left) and southern California (right) watersheds

The same pattern was observed in the amount of modeled sediment exported from the land covers before and after the fire event. In the Mark West subwatershed, an increase of 49,961 tons of sediment was simulated in the post-fire SDR scenario, indicating a rise in sediment export induced by the fire event. The modeled spatial difference in sediment export for the Mark West subwatershed ranges between 0 and 94 tons ha^−1^ year^−1^, while that of southern California sub-watershed extends from 0 to 139 tons ha^−1^ year^−1^ as shown in Fig. 4.

### Carbon Storage and Sequestration

The results for Total Above Ground Carbon and other categories are found in Table 4. Both fires resulted in large losses in total above ground carbon. According to our modeling, the Tubbs fire resulted in a loss of 199,318.57 tons of carbon from the Mark West subwatershed. The Thomas Fire’s impact was smaller and across a relatively larger land area. A total of 158,662.41 tons of carbon were lost from the South Ventura, Harmon Canyon, and Arrundell subwatersheds. Together, the two fires resulted in an emission of 1,312,597 tons of CO_2_ equivalent carbon. For context, annual US GHG emissions are about 6 billion tons of CO_2_ equivalent carbon.

**Table 4:** Changes in total above ground carbon stores at the watershed level

Carbon stores (in tons)	Mark West	Southern California
Canopy	−30,160.41	−52,545.93
Shrub	−19,432.82	−12,1875.72
Herb	−2,393.51	−294.13
Wood	−67,473.64	−2,062.04
Litter Layer Mass	−6,994.54	−81,812.24
Ground	−71,878.7	−5,181.01
Total Above Ground	−199,318.57	−158,662.41

### Nutrient Delivery

**Fig. 5:**
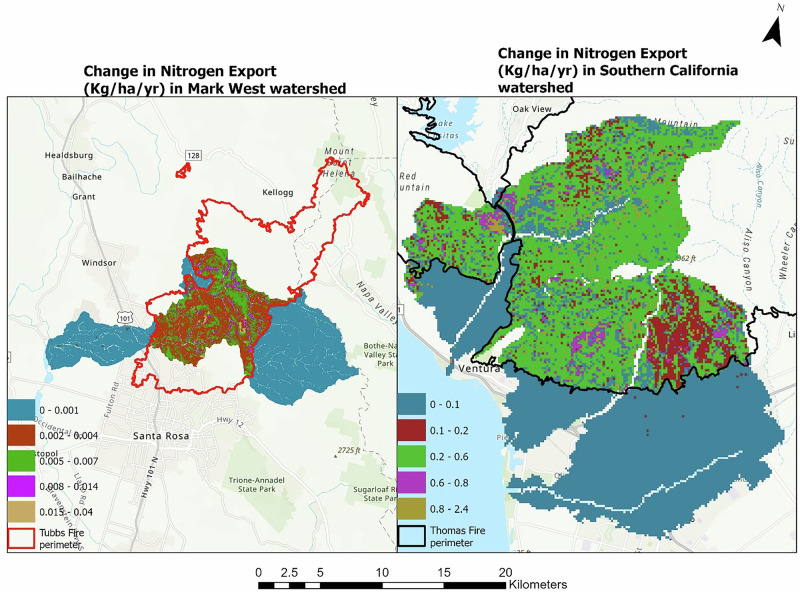
The figure shows the difference in nitrogen export (kg ha^−1^ year^−1^) before and after the fire in the Mark West (left) and southern California watersheds (right)

Mean estimated Nitrogen export in the Mark West subwatershed was 0.012 kg ha^−1^ year^−1^ before the fire and 0.014 kg ha^−1^ year^−1^ after the fire; a substantial 16.7% increase. The modeled spatial difference in Nitrogen exports in this same watershed ranges between 0.001 kg ha^−1^ year^−1^ to 0.04 kg ha^−1^ year^−1^ as shown in Fig. 5. Nitrogen exports in the southern California sub-watersheds showed similar trends as the Mark West counterparts, with increased nitrogen exports after the fire event. The modeled mean difference in the nitrogen export before and after the fire in the watershed in Southern California watershed was 0.18 kg ha^−1^ year^−1^ and ranged between 0.001 and 2.34 kg ha^−1^ year^−1^ spatially as shown in Fig. 5. An 11% increase in modeled total Nitrogen export was observed in the Mark West subwatershed while about 14% increase was found in the southern California watershed.

**Fig. 6:**
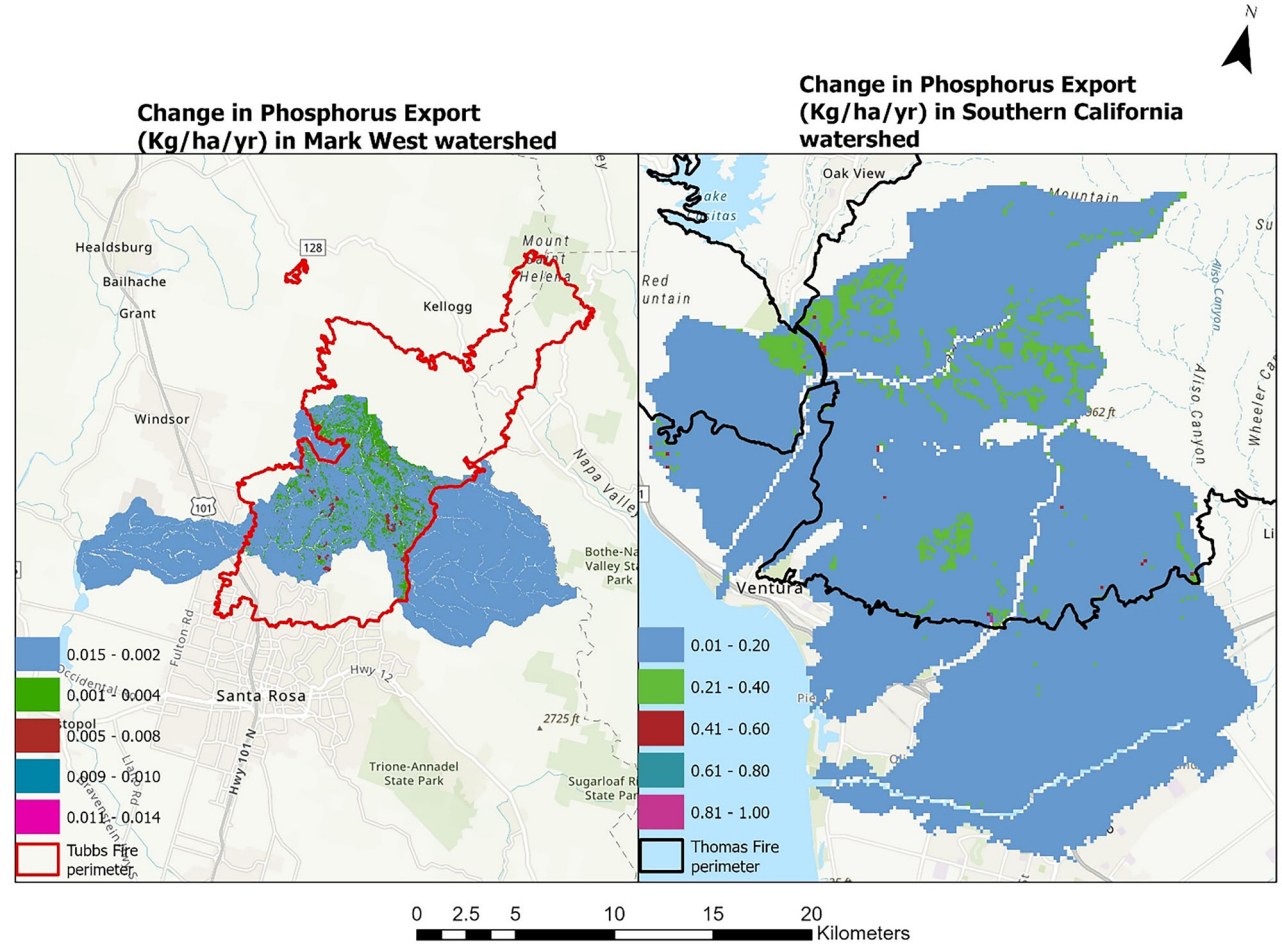
The figure shows the difference in phosphorus export (kg ha^−1^ year^−1^) before and after the fire in the Mark West (left) and southern California watersheds (right)

Phosphorus exports in the watersheds also increased in both watersheds, according to our models. The total phosphorus exports in the southern and northern California watersheds before the fires were 5058 Kg and 1248 Kg, respectively. These values increased by 20% and 16% in the southern California and Mark West watersheds respectively as shown in Tables 2, 4. The spatial difference in phosphorus exports ranges between 0 and 0.916 kg ha^−1^ year^−1^ in southern California sub-watersheds and 0 and 0.013 kg ha^−1^ year^−1^ in the Mark West subwatershed as shown in Fig. 6.

### Impacts of Wildfire to Communities

In general, we found that the study communities’ demographic variables do not have a substantial correlation with the distribution of the pre-fire ecosystem service supply estimates from InVEST except in two distinct cases. Specifically, US census blocks that had higher levels of linguistic isolation were related to lower levels of annual pre-fire sediment loss, phosphorous loading, and nitrogen loading (Tables 5, 6). Interestingly, linguistic isolation was not related with the pre-fire level of water runoff.

**Table 5:** Regression results for estimating the impacts of sociodemographic variables on the pre-fire levels of water yield, sediment loads, phosphorus, and nitrogen. All variables are transformed using the hyperbolic arcsine transformation, and the coefficients can be regarded as approximations of percent changes

Sociodemographic variables		*Dependent variable:*		
	Pre-fire water yield	Pre-fire soil loss	Pre-fire phosphorus	Pre-fire nitrogen
	(1)	(2)	(3)	(4)
Unemployment	−0.141*	0.051	0.010	0.019
	(0.078)	(0.091)	(0.006)	(0.015)
Poverty	−1.038***	−0.005	0.022	0.074
	(0.325)	(0.380)	(0.027)	(0.065)
Housing Burden	−0.370**	−0.021	0.003	0.032
	(0.158)	(0.183)	(0.012)	(0.030)
Education	0.370***	−0.004	−0.008	−0.030
	(0.108)	(0.125)	(0.008)	(0.021)
Linguistic Isolation	−0.038	−0.140**	−0.012**	−0.024**
	(0.055)	(0.064)	(0.005)	(0.011)
Constant	9.206***	1.576	0.011	−0.039
	(1.351)	(1.577)	(0.110)	(0.269)
Observations	63	63	60	60
R^2^	0.247	0.087	0.169	0.166
Adjusted R^2^	0.181	0.007	0.092	0.089

*p < 0.1, **p < 0.05, ***p < 0.1

**Table 6:** Regression results estimating the impacts of sociodemographic variables on the change in ecosystem services following a fire for water yield, sediment load, phosphorus, and nitrogen. All variables are transformed using the hyperbolic arcsine transformation, and the coefficients can be regarded as approximations of percent changes

		*Dependent variable:*		
	Difference in water yield	Difference in soil loss	Difference in phosphorus	Difference in nitrogen
	(1)	(2)	(3)	(4)
Pre-fire water yield	−0.444*			
	(0.256)			
Pre-fire sediment load		0.095		
		(0.120)		
Pre-fire phosphorus			0.306***	
			(0.026)	
Pre-fire nitrogen				0.240***
				(0.033)
Unemployment	0.094	0.042	0.0001	0.003
	(0.156)	(0.083)	(0.001)	(0.004)
Poverty rate	−0.193	0.515	−0.003	−0.011
	(0.682)	(0.345)	(0.005)	(0.016)
Housing burden	0.478	0.284*	−0.004	−0.013*
	(0.319)	(0.166)	(0.002)	(0.007)
Education	−0.239	−0.219*	0.001	0.003
	(0.230)	(0.114)	(0.002)	(0.005)
Linguistic isolation	0.021	−0.010	−0.001	−0.004
	(0.107)	(0.061)	(0.001)	(0.003)
Constant	3.992	−2.215	0.014	0.054
	(3.517)	(1.444)	(0.021)	(0.066)
Observation	63	63	60	60
R^2^	0.142	0.123	0.771	0.567
Adjusted R^2^	0.05	0.029	0.745	0.517

*p < 0.1, ***p < 0.1

Water runoff, in turn, was negatively associated with unemployment, poverty, housing burden, and education, all of which are statistically significant at the 5% level, however it was positively correlated with US census blocks with higher education (Table 5; Column 1). It is important to note that several of the results might be driven by topographical features inherent to the communities (e.g. slope). We then estimated Eq. (3), which included the pre-fire levels of each InVEST variable to obtain Table 6.

Many of the differences between the InVEST variables before and after the fire were correlated with their pre-fire levels. For instance, the pre-fire levels of water yield were negatively correlated with changes in water yield following a fire ($\beta =-0.444$) such that the more pre-fire water yield there was, the lower the change due to wildfire was. Similarly, pre-fire levels of nitrogen and phosphorus loading were positively correlated with the change in nitrogen ($\beta =0.240$) and phosphorus ($\beta =0.306$) following a fire. Thus, the more pre-fire nitrogen and phosphorus there was, the higher the difference between pre- and post-fire levels of nitrogen. The pre-fire level of soil loss before the fire was not statistically significant in the regression.

Overall, there were only three instances in which any of the socio-demographic variables were related to ecosystem service outcomes. First, soil loss was positively correlated with housing burden. Second, soil loss was negatively correlated with education. Third, housing burden was negatively correlated with nitrogen loading.

## Discussion

The large increase in water yield occurring within the southern California watersheds in the model is consistent with the large and deadly debris flows that occurred following the Thomas Fire (Addison and Oommen 2020). The differences in results between watersheds was likely driven by differences in landscape type. The southern California watersheds are typically chaparral landscapes, which might have contributed to this difference, since chaparral landscapes might have particularly higher post-fire potential for increased runoff (Hubbert et al. 2006). One caveat is that while our analysis showed the change in annual water yield, the change in seasonal water yield might be different. For example, Adamowicz et al. (2019) showed that forests can better store water and provide it during the dry season. Our results are consistent with that of different studies such as Saxe et al. (2018) and Blount et al. (2020) who also show an increase in annual water yield because of a fire event.

The rise in water yield observed in the southern California watersheds is also consistent with those observed by Kinoshita and Hogue (2015) in their assessment of the impact of the 2003 Old Fire event on two ephemeral watersheds in southern California. The mean actual evapotranspiration in the watershed was also reduced from 844 mm in the pre-fire scenario to 700 mm in the post-fire scenario. Vegetations such as trees and shrubs are often characterized by taking up a significant amount of water through transpiration and interception (Turner 1991). However, in the event of wildfire, as in this study, vegetation loss could lead to increased water yield in the watershed (Basso et al. 2020; Pereira et al. 2016). In addition, reduced evaporation from soils and other surfaces could increase streamflow into rivers and potentially increase water yield (Kinoshita and Hogue 2015). Similar to our study, Blount et al. (2020) reported a post-fire 140% increase in water yield, associated with conversion of forest to post-fire shrubland and grasslands. The model-simulated post-fire water yield showed dynamics similar to the observed discharge (five-year post-fire average) from nearby USGS gauging stations, with both studied landscapes exhibiting increased discharge (Table S2). However, the 3% increase in post-fire precipitation may have also contributed to the observed discharge increase.

There is a considerable amount of wildfire science research that has examined how wildfire impacts water yield. Results from this body of work suggest that impacts can be mixed depending on factors such as fire severity, post-fire precipitation and fire history. For instance, forest fire events may affect soil permeability by reducing soil compaction, allowing water infiltration into the soil, and increasing yield as baseflow (Gonzalez-Romero et al. 2021). Other work shows that wildfire can increase the repellency of soils, resulting in more water yield (Hubbert et al. 2006). Fire also affects soil hydraulics by depositing ash and sediments and developing water repellent CSIRO (2012), which could lead to increase in water yield Moody and Martin (2001b). The long-term increases in water yield resulting from severe and widespread fires could modify the functioning of riparian ecosystems (Salemi et al. 2012). However, it may also offer a distinct possibility to supplement the regional water supply used by urban areas and agricultural communities.

We also found an increase in the expected soil loss and erosion across both the Mark West subwatershed and the southern California watersheds. While we provided the justification for the increase in soil loss and erosion based on the modeling approach, in practice, several other factors could also drive the increase in soil loss after a fire. For example, fires can reduce the ability of the soil to hold moisture by burning organic matter, making the soil more erodible (Shakesby and Doerr 2006). Soil structure disruption associated with intense fire heat increases soil compaction and reduces infiltration capacity, making the soil prone to erosion. Post-fire activities also lead to increased surface runoff and high flow with the potential of detaching and transporting significant soil particles. Ash deposition and soil heating can lead to a hydrophobic layer on the soil, reducing water infiltration into the soil, which could result in high surface runoff and favor the removal of soil particles (Larsen et al. 2009). Alteration of channel morphology, such as increased channel roughness and formation of sediment deposits after a fire event associated with burnt land cover, could also enable higher sediment export (Moody and Martin 2001b). Fires can have a severe impact on channel morphology which influences sediment availability. Channels provide sediment through the upstream extension of head cuts, lateral bank erosion, and further destruction of banks (Moody and Martin 2009).

Erosion rates and sediment yield have been widely documented to increase after fire events, often due to the direct and indirect effects of the burning (Moody and Martin 2001a; Robichaud et al. 2020; Biswas et al. 2021). Other studies also report an increase in sediment yields as a result of wildfires. For example, East et al. (2021) showed that post-fire sediment yield in Whiskeytown National Recreation Area in Northern California increased after the Carr Fire. In another study, using a physically based model Rulli and Rosso (2005) found a substantial increase in erosion and sediment in nine basins in southern California due to a series of wildfires. Finally, Coombs and Melack (2013) found an increase in suspended sediment export from 82% burnt San Onofre watersheds characterized by chaparral vegetation in southern California compared to similar unburnt watersheds.

The rise in soil loss and sediment export could be related to vegetation loss induced by fire events (Parise and Cannon 2012). This is caused by the loss of plant roots and above-ground biomass, which removes the protective cover that holds the soil and makes it susceptible to gradual soil loss. Forest and vegetation covers are important for binding soil particles together using a network of fibers in their roots which increases soil stability and prevents erosion (Parise and Cannon 2012). The loss of above-ground biomass, such as leaves, stems, and branches, exposes the soil to the direct impact of rainfall and wind, which triggers the loss of soil particles. The loss of canopy cover in forested watersheds due to fire events reduces rainfall interception and increases the wind effect on the soil. Hanshaw et al. (2009) observed that rainfall amount measured by a rain gauge beneath a chaparral shrub canopy was reduced to about 42% than those observed in adjacent burnt areas. These actions in isolation or combination can increase the pressure on the soil surface and their erosive potential. Furthermore, vegetation loss due to wildfire could reduce the stabilization of slopes and increase soil erosion along the slopes due to landslide mass movement and increase the rate of sediment exportation (McEachran et al. 2021). However, as previously mentioned, changes may depend on the severity of the fire and various aspects of the landscape affected (Keely et al. 2006).

The differences in the carbon losses reflect the differences in the size of the watersheds and the differences in land cover types across regions. For instance, while the Mark West lost 19,432.82 tons of carbon in the shrub category, the southern California Watersheds lost 121,875.72. This represents approximately 0.3% of the 45 teragrams of total carbon storage in the southern California National Forests reported by Underwood et al. (2019). Similarly, the southern California Watersheds lost far more carbon in the Litter Layer Mass (LLM) category. The Mark West subwatershed lost substantially more carbon in the wood category (67,473.64 tons) than the southern California sub watersheds (2062 tons). Interestingly, though the Mark West subwatershed lost 30,160 tons of carbon in the Canopy category, the three southern California watersheds gained 52,545 tons in the year following the fire. This is likely due to the pattern of landscape changes that occur following a fire for fire-adapted chaparral ecosystems (Storey et al. 2021).

**Fig. 7:**
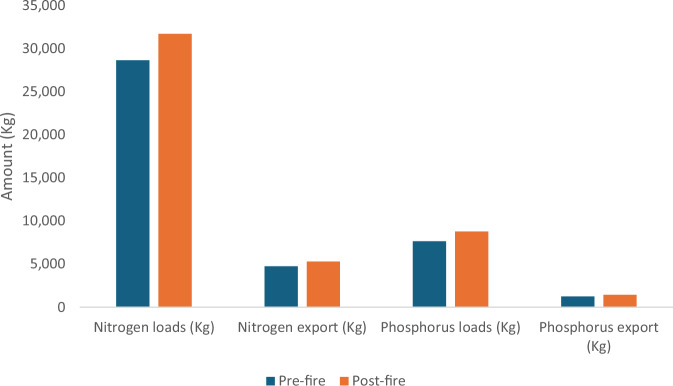
Nutrient dynamics in Mark West watershed

**Fig. 8:**
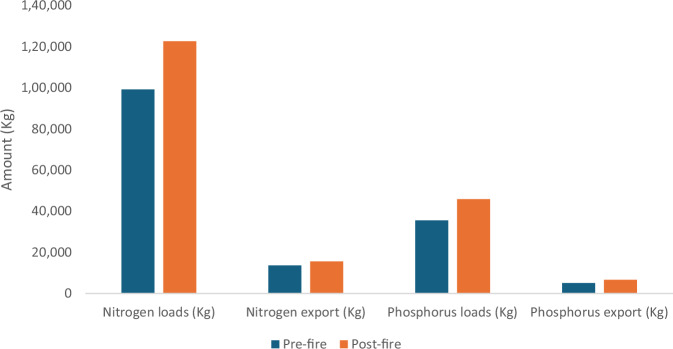
Nutrient dynamics in southern California watershed

Our analysis showed an increase in the expected post-fire phosphorus and nitrogen export in both studied watersheds (Figs. 7, 8). Increased phosphorus and nitrogen exports in the watersheds could be detrimental to downstream waterbodies by providing conditions that favor the rapid growth of algal and other invasive species (Brehob et al. 2024), although they could be favorable for downstream oligotrophic aquatic ecosystems (Rogora et al. 2021). Comparing the nutrient exports with other studies from different study areas could be complex due to varying rainfall amounts triggering runoff generation, the variation in fire severity, post-fire activities, ecosystem characteristics that drive nutrient mobility, and variation in methods used for nutrient measurements (Lane et al. 2008; Smith et al. 2011). However, studies such as Ferreira et al. (2005), Coombs and Melack (2013) and Goodridge et al. (2018) also showed an increase in nutrient exports after a fire event. Coombs and Melack (2013) specifically observed increased dissolved organic nitrogen (DON) and phosphorus exports in a burnt chaparral-dominated watershed in southern California triggered by a high storm event and additions of Nitrogen in the form of ammonium and DON deposited as ash on the soil surface. Similarly, McCullough et al. (2023) reported increased in median concentration of total nitrogen (68%) and total phosphorus (70%) following the Greenwood fire in Minnesota, USA. Elevated TN and TP was also observed in a recent study by Raoelison et al. (2023) which showed that the increase in the nutrient concentration is often significant during post-fire storm events, especially during first flush which is associated with when solute concentrations reach their maximum (Gallaher and Koch 2004). Nutrient concentrations are often highest within first year of wildfire, then decrease with time to background levels a couple of years after the fire (Arkle and Pilliod 2010).

Increased nitrogen and phosphorus exports in burnt watersheds can be related to the loss of vegetation cover (Rodríguez-Romero et al. 2018). Vegetation cover plays a vital role in the uptake of nutrients from the soil. Their loss could lead to decreased nutrient retaining capacity and increased availability of these nutrients for export. Soil disturbance associated with burnt landscapes interferes with erosion intensity and soil compaction, which disrupts natural nutrient cycling and could lead to increased availability of nitrogen and phosphorus to be exported Smith et al. (2011). The enhanced export of nitrogen and phosphorus from an ecosystem can also be considerably influenced by ash deposition after a fire (Goforth et al. 2005). Ash introduces a variety of organic and inorganic components, such as nitrogen and phosphorus, when deposited on the soil’s surface. As a result, many mechanisms that improve nutrient export are implemented (Reneau et al. 2007). Nutrients are easily freed from ashes, making them easier to distribute; however, InVEST does not consider this effect.

On a regional basis, the increase in post-fire nitrogen and phosphorus export observed in this study aligns with the observed trend in the western United States, often associated with elevated phosphorus and nitrogen levels during the first five years after a fire event (Rust et al. 2018). These increases in nutrient exports might have stringent implications for water quality and management strategies.

In terms of wildfire impacts to communities, we found that differences in modeled water yield, soil loss, phosphorus, and nitrogen levels before and after wildfire were largely not correlated with the sociodemographic characteristics. However, the pre-fire levels of these variables were correlated with sociodemographic variables. The implication of these results is that although ecosystem services are unevenly distributed across the landscape, wildfires neither exacerbated nor improved the distribution of ecosystem services, according to our modeling. It is important to note that this result might be specific to the: specific ecosystem services considered in this study, analysis period, and socio-ecological contexts of the two communities we studied.

When estimating Eq. (2), several relationships were found between ecosystem service provision and the sociodemographic characteristics of the communities. For instance, a 1% increase in the poverty rate of a census tract was correlated with a 1.038% decline in water yield, such that the higher the poverty rate of a census tract, the less water was flowing through the census tract. This observation was also true of housing burden, where a 1% increase in housing burden was correlated with a 0.37% decline in runoff, all else constant. Recent work has examined linkages to water and poverty levels. For instance, Alqatarneh and Al-Zboon (2022) created a water poverty index for resource management in Jordan. In the United States, Deitz and Meehan (2019) investigated hot spots for uneven water supply along racial and geographic lines, finding clear relationships between the quality of plumbing and race. The result of our study aligns with the findings of Deitz and Meehan (2019) in identifying distributional unevenness in water resources, even in wildland systems. This highlights the need for tools like those presented in Alqatarneh and Al-Zboon (2022) for helping to address these issues. However, while existing literature suggests poverty mediates access to natural resources, our findings do not directly evaluate this relationship.

We also found that linguistic isolation is negatively correlated with soil loss, such that a 1% increase in linguistic isolation is correlated to a 0.14% decline in soil loss. There are similar relationships between linguistic isolation and phosphorus and nitrogen loading; however, the magnitudes of the changes are exceptionally small. Thus, our results indicated that in general, pre-fire levels of soil loss are lower in communities with fewer English speakers. Similar studies such as Masri et al. (2020) generally focus on soil quality rather than soils loss; and in this instance, they found that soil lead distributions in the city of Santa Ana, California, was much higher in socioeconomically disadvantaged urban neighborhoods.

Though many studies have addressed how wildfires impact the provision of ecosystem services, (Lee et al. 2015; Vukomonovic and Steelman 2019; Pereira et al. 2021), to our knowledge there is no other study that addresses the distributional impacts of wildfires on ecosystem service provision to communities. However, the distributional equity of ecosystem service supply has been a well-studied issue. Much of the work on ecosystem services and socio-demographics characteristics of the beneficiaries has been done in urban ecosystems (e.g. Geneletti et al. 2020) with others expanding analysis to the WUI (e.g., Thomas et al. 2022; D’Evelyn et al. 2022). Our results showed that the unevenness in the distribution of water, soil loss, nitrogen, and phosphorus loading are mainly driven by pre-existing conditions that were present prior to wildfire. There are also many other dimensions of the direct and indirect impacts to communities from wildfire, including who is impacted by home loss and smoke (Yadav et al. 2023). Future work might benefit from comparing these direct and indirect effects before and after fires and other large natural disasters to see how these events impact different communities (Thomas et al. 2022). Such work will be vital for identifying changes to policy needed to alleviate issues of sustainability and equal access to ecosystem services.

Overall, we found little evidence for a community’s sociodemographic characteristics being correlated with either pre-fire ecosystem service levels or with differences in ecosystem service levels post- fire. There is ample literature that seeks to illustrate how sociodemographic groups might be affected differing levels of environmental quality (e.g. Martín-López et al. 2012; Faccioli et al. 2020). However, one way to interpret our findings is that we do not find evidence to support this literature, at least in the watersheds which we included in this study.

## Conclusion

We assessed the effects of wildfires on annual water yield, carbon sequestration, soil loss, sediment exports, phosphorus delivery, and nitrogen delivery. We found that even though changes in nitrogen were similar, the southern California watersheds experienced a change in phosphorus almost twice as large as that in the Mark West subwatershed. In addition to assessing the changes in magnitudes of ES pre- and post-fire, we also examined how ESs are distributed across communities pre- and post-fire. Where most studies examining impacts of wildfires on regulating ecosystem services look at temporal changes, our study connects these changes to community level distributions of ESs in order to examine the impacts of wildfires across different socio-demographic groups. In our study region, we found that while ESs are distributed unequally pre-fire, the fire does not have a statistically detectable impact on either exacerbating or alleviating inequalities that existed before the fire.

The differences and similarities in how fire affected each region is important for post-fire management. Importantly, our modeling results were consistent with findings from Hubbert et al. (2006) in that chaparral landscapes have particularly high potential for runoff events following fire compared with non-chapparal landscapes; even leading to disservices in the form of floods. Consistent with Hubbert et al. (2006), even though the change in post fire runoff is large compared to pre fire scenario, changes in sedimentation, nitrogen, and phosphorus levels of the water are not high.

There are several important limitations and avenues for future research. A relevant limitation of the annual water yield model for this study is the lack of detailed spatial land cover data so that complex land use changes can be adequately characterized. This limitation means that complex changes in land use may not be comprehensively accounted for in model predictions (Sharp et al. 2020). The InVEST model was selected due to its flexibility, interpretability, and reproducibility; however, other modeling frameworks might provide more accurate results for focused studies, such as Soil and Water Assessment Tool. Further, there are a wide variety of other ecosystem service bundles that could be considered. We limited our analysis to regulating ecosystem services due to the salience and importance in the California context; however, cultural ecosystem services like recreation, and ecosystem disservices are also important to consider by future research. Past studies on the topic have also considered the economic values of these ecosystem services (e.g. Underwood et al. 2019). Future work can combine flexible benefit transfer methodologies with modeling output to achieve estimates of economic damage resulting from the fire. However, this might be challenging, especially for water resources, since the per-unit values of water resources might be dependent on the volume. Moreover, we did not consider fire effects on hydro-power generation. Future work can consider the additional costs of fires on production outcomes, such as electricity.

We investigated the role of wildfires in changing the way in which ecosystem service benefits are distributed across different communities and watersheds. In general, we found that even though benefits from ecosystems in the form of water resources and carbon sequestration, are distributed differently across different US census blocks, that wildfires did not have any statistically significant impacts on those distributions. We found that this was consistent across different types of landscapes. There are several areas of future research that our results highlight. Future work can benefit from incorporating the community's adaptive capacity into post-wildfire recovery. The relationship between natural disasters and the provision of ecosystem services is a well-studied topic that still commands attention from various disciplines. As wildfires continue to affect human communities more, understanding their impacts will become more necessary.

## Supplementary information

Supplementary_Ecosystem_Service_4_14_25

## Data Availability

No datasets were generated or analysed during the current study.
