# Supplementary material for: Modeling Wildfire Effects on Ecosystem Services in two Disparate California Watersheds and Communities
Source: Environ Manage. 2025 May 23;75(7):1680–700. doi: 10.1007/s00267-025-02185-3 (PMC12228666; doi:10.1007/s00267-025-02185-3)
Supplement: Supplementary file 1 — Supplementary_Ecosystem_Service_4_14_25 [file 267_2025_2185_MOESM1_ESM.docx]

**Land Cover Change Analysis**

We first examined the impacts that the Tubbs and Thomas fires had on the landcover distributions of the Mark West and Southern California Watersheds, respectively. We broadly categorized the land cover categories from the fire into four categories. We calculated the sums before and after the fire and present them in Table S1.

**Table S1**: Percent change in land cover after Tubbs and Thomas fires for the Mark West and Southern California Watersheds.

| Land cover | Mark West | Southern California |
| --- | --- | --- |
| Forest | -0.38% | -17.33% |
| Woodland | -1.43% | -11.12% |
| Grassland | 0.17% | 10.59% |
| Chaparral | -5.93% | -41.10% |

The same patterns emerged from both the Mark West watershed as well as the Southern California watersheds; albeit in different magnitudes. In all cases, a year following fire, the amount of forestland, woodland, and chaparral declined; however, the amount of grassland increased. In the Mark West, there was 0.38% less forest following the fire, 1.43% less woodlands, and 5.93% less chaparral. The quantity of grasslands increased by only 0.17%. In Southern California, the changes were much larger in percentage terms. The quantity of forests declined by 17.3%, woodlands by 11.12%, and chaparral by a staggering 41.10%. Grasslands increased in southern California by 10.59%. It is important to note that the percentages may vary based on the previous level of land cover prior to the fire.


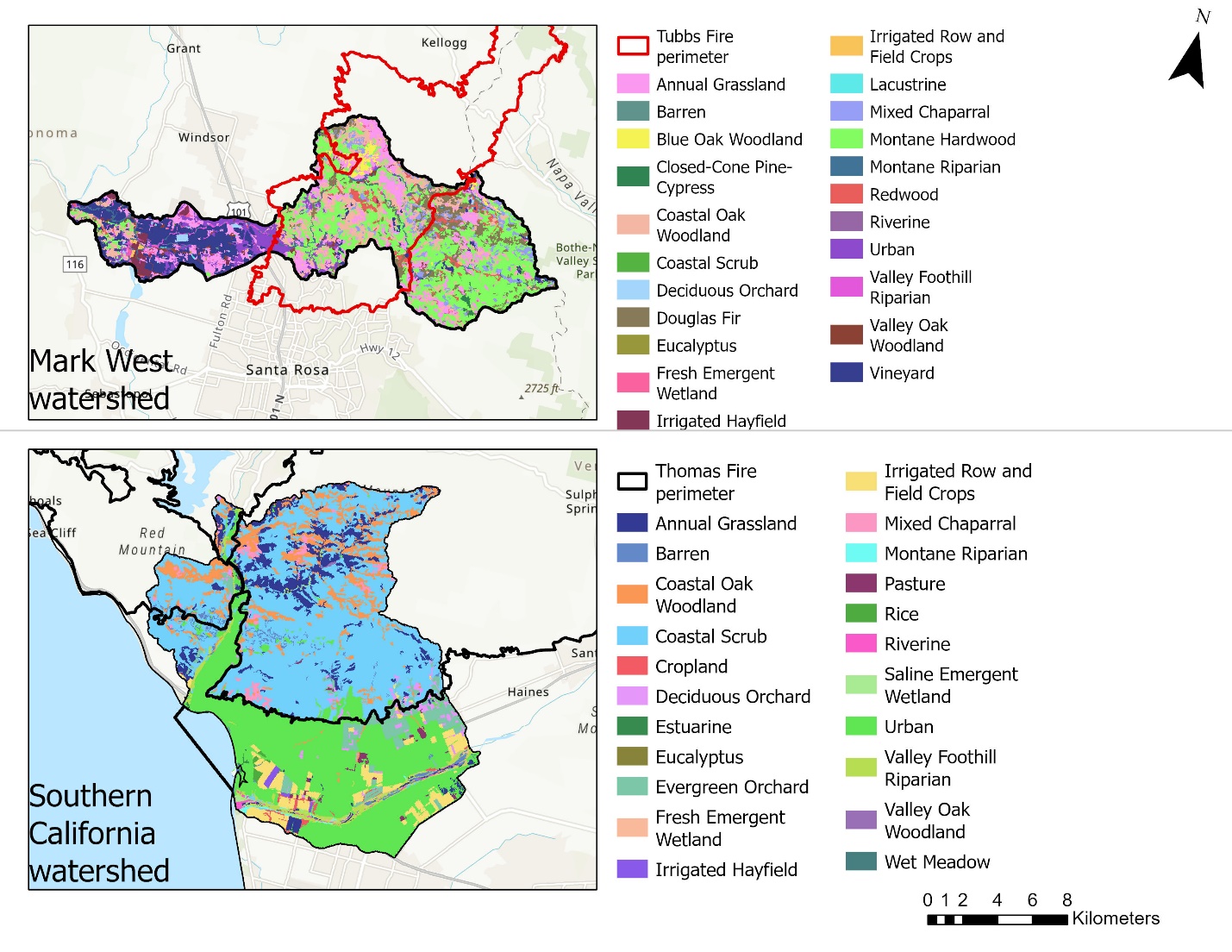


**Fig. S1.** Map of Study Area showing vegetation cover

**Table S2:** Discharge for Southern California and Mark West watersheds for the pre-fire and post fire periods according to USGS 11118500 and 11466800.

Note: Pre-fire= Averaged over 5 years before the fire, Postfire=Averaged over 5 years after the fire).

| **Watershed** | **USGS Gauge ID** | **Pre-fire discharge (cf/s)** | **Postfire discharge (cf/s)** | **USGS**  **%Increase** | **Modeled**  **%Increase** |
| --- | --- | --- | --- | --- | --- |
| Southern California | 11118500 | 10.24 | 12.10 | 18.12% | 34% |
| Mark West | 11466800 | 202.57 | 206.83 | 2% | 6% |

**Fire Descriptions**

*Tubbs Fire*: The Tubbs fire started on October 8th, 2017, near Bennet Lane in Napa County and burned approximately 36,810 acres (14,897 hectares) in three counties before reaching final containment on October 31 2017. The fire was sparked by downed electric utility lines that were overwhelmed by high winds (sustained gusts up to 68 mph). Pushed by the high velocity easterly winds, the Tubbs fire moved rapidly through the Maycamas Mountains traveling 12 miles in roughly 4 hours as it burned through sparsely populated landscapes and into Santa Rosa city limits. The Tubbs Fire was California’s most destructive wildfire until the Camp Fire one year later. Twenty-two people are believed to have lost their lives in the Tubbs fire with 5,643 structures destroyed (LeComte, 2018; Cortenbach et al., 2019).

*Thomas Fire:* The Thomas fire started one month later on December 4th, 2017, and was not declared contained until January 12, 2018, and officially declared out on June 1 2018 after months of inactivity. The Thomas Fire was much larger than the Tubbs fire burning 281,893 acres (114,078 hectares) in Santa Barbara and Ventura Counties. For a short time, the fire was the largest in California history and currently ranks as the seventh largest wildfire recorded in the state. The Thomas fire was also pushed by high Santa Ana winds with gust up to 70 mph and moved from its start near Thomas Aquinas College into Ventura burning 500 homes within the city limits the first night. At its close, the Thomas fire burned roughly 1,343 structures and caused over $2.2 billion in damages (Lukashov et al., 2019; Kolden and Henson, 2019; Kress, 2020).

**Percent of Mark West Watershed burned by Tubbs Fire**.

- Tubbs Fire extent: 14,876.0 ha (36,759.4 acres)
- Tubbs Fire in Mark West watershed: 5,636.3 ha (13,927.6 acres); 38% of Tubbs Fire Footprint in Mark West watershed
- Mark West watershed: 13,710.2 ha (33,878.6 acres)
- Mark West watershed burned by Tubbs 5,636.3 ha (13,927.6 acres) 41% of Mark West watershed

**Percent of Southern Californian Watersheds burned by Thomas Fire.**

- Thomas Fire extent: 114,036.7 ha (28,1790.9 acres)

Area covered by the three watersheds burned in Thomas Fire

- Harmon Canyon-Santa Clara watershed: 10,080.3 ha (24,909 acres)
- Arundell Barranca-Frontal Pacific Ocean: 5,656.6 ha (13,977.6 acres)
- Lower Ventura River: 10,593.1 ha (26,176 acres).

Thomas Fire footprint in the three southern California watersheds:

Total Area = 14018.3 ha; 26333 acres. 53% of the three watersheds listed below

- Harmon Canyon-Santa Clara watershed: 2329.3 ha; 5755.8 acres. 23% of Harmon Canyon-Santa Clara watershed
- Lower Ventura River watershed: 8513 ha; 21036.1 acres. 80% of Lower Ventura Watershed
- Arundell Barranca-Frontal Pacific Ocean watershed: 3176 ha; 7848.1 acres. 56% of Arundell Barranca-Frontal Pacific Ocean watershed

**Fire Return Intervals**

*Tubbs Fire*

**Pre-Euro American Settlement:** Vegetation within the Mark West watershed and surrounding areas is comprised of a mix of conifer forests (mostly redwoods), mixed conifer hardwoods (Douglas Fire & assorted evergreen oak species), shrublands (chaparral and coast sagebrush), grasslands, and vineyards (see FRID discussion).

**Dendroecology studies:** Several dendrochronological studies using tree ring data from conifers to date past fires have been produced within the Mark West watershed (Finney and Martin 1992) and surrounding areas (Finney and Martin 1989, Skinner et al 2009). These studies provide evidence that the Tubbs Fire area burned much more frequently before white Euro-American settlement than the current, burn pattern. Finney and Martin (1992), working within the Annadel State Park which burned during the Tubbs Fire, reported a fire return interval of 6 – 23 years. This record of burning was collected from the tree rings of 14 redwood stumps that were logged before 1866 prior to the establishment of Annadel State Park. Earlier, Finney and Martin (1989) collected tree ring data from nearby Salt Point State Park, approximately 50 west of Annadel State Park, indicating that more coastal area had burned at a fire return interval of 20 to 29 years pre-Euro American settlement. Additional information to support the premise of fire burning much more frequently in the past was provided by a Skinner et al. 2009 fire history study in the Mendocino National Forest where the authors reported a fire return interval of 4 to 38 years.

**Historical Fires:** Our spatial data from the historical CalFire FRAP fire history data set (https://www.fire.ca.gov/what-we-do/fire-resource-assessment-program) indicates that large wildfires such as the Tubbs have occurred periodically in the 20th and 21st centuries (Fig. S2) in north central coastal California. The FRAP data records three fires prior to the 2017 Tubbs Fire within the Mark West Watershed: The 1964 Hanly Fire (burned 5163 ha (12758 acres) in Mark West Watershed; 38% of watershed; 92% of the Tubbs fire area), the 1996 Porter Creek Fire which burned 105.7 ha (261.2 acres), and the 2004 Oceguera Fire 7.9 ha (20 acres) (Fig. S2).

After the 2017 Tubbs Fire, the general area surrounding Mark West has burned in 5 different wildfire events 2019 Kincade Fire (336.5 ha, 831.5 acres), 2020 Glass fire (3.7 ha, 9.1 acres), 2020 Usuline (3.1 ha, 7.7 acres), 2020 Creek Fire (0.5 ha, 1.2 acres), and the 2021 West Fire (1.0 ha, 2.5 acres). Kincaide, Glass, and Creek Fires were larger fires that burned a short way into the Tubbs Fire footprint while the Creek and West fires were small fires that ignited with the Tubbs Footprint and went out or were suppressed.


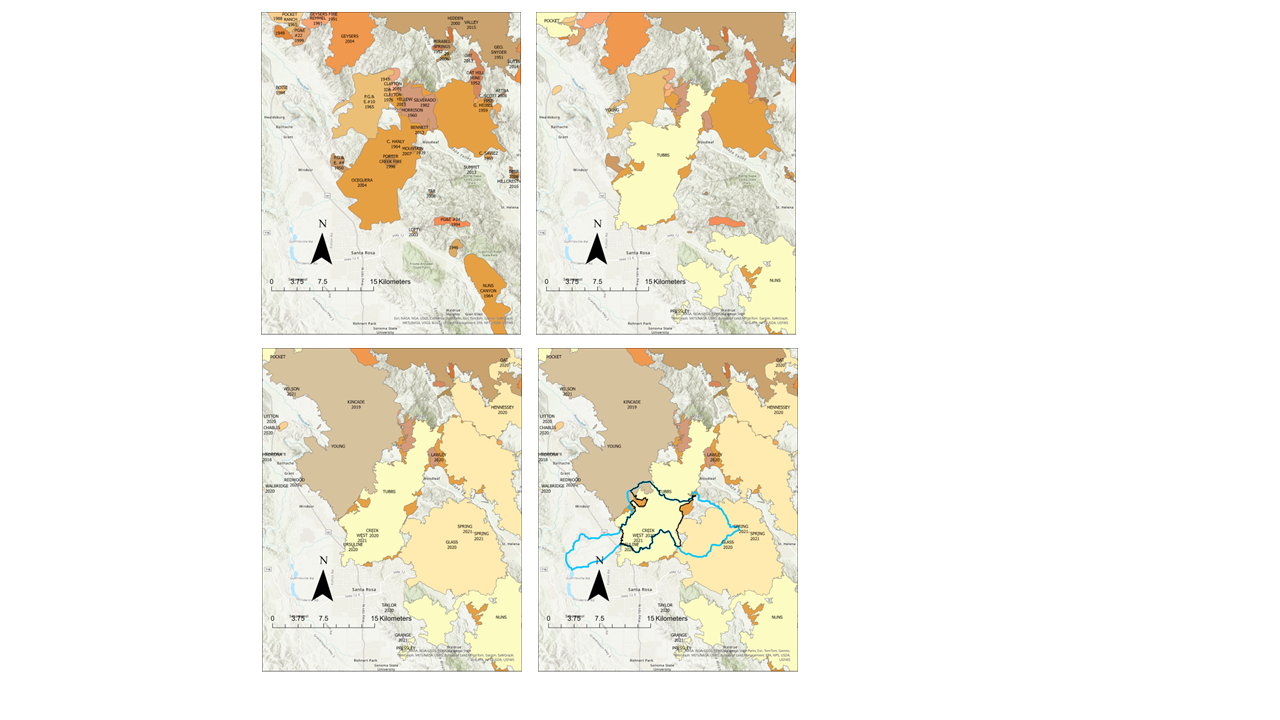


**Fig. S2.** Spatial Fire History in Tubbs Fire area 1910 – 2022. Sonoma County Ca. Panel A. 1910 to 2016 Wildfires. Panel B. 2017 Wine Country Fires Tubbs, Nunns, and Pocket fires. Panel C. Fires 2018 through 2022. Panel D. Mark West Watershed boundary (blue). Mark West/ Tubbs Fire intersection.

*Thomas Fire*

**Pre-Euro-American Settlement**: Vegetation within the Thomas Fire burn area is largely comprised of shrublands predominately classified as coastal sage brush and chapparal (see FRID discussion in this supplement). As such, evidence regarding the frequency and extents of pre-Euro-American settlement fires is sparse due to the lack of forest vegetation to collect dendrochronological (tree ring) evidence of past fires. However, evidence from other sources including sediment cores from the Santa Barbara Basin indicate that Pre-Settlement fires were similar in size to recent fires in southern California Shrublands (Mensing et al 1999, Keeley and Fotheringham 2001, Keeley and Brennan 2012, Syphard et al. 2007, 2019). What does appear to have changed is the interval between successive fires, current large wildfires such as the Thomas Fire are occurring more frequently than in the past (Mensing et al 1999, Keeley and Fotheringham 2001, Keeley and Brennan 2012, Syphard et al. 2007, 2019). Moreover, the increased frequency of large fires appears to be driving a type conversion from shrublands to more flammable grasslands that are likely to burn even more frequently (Syphard et al. 2007, 2019).

**Historical Fires:** Our data from the historical data set (CalFire FRAP https://www.fire.ca.gov/what-we-do/fire-resource-assessment-program) supports this viewpoint (Fig. S3). Large fires occurred across this landscape before the Thomas Fire during the 20th and early part of the 21st century – most notably the 1929 Sulphur Mountain Fire (10279 ha), the 1932 Matilda Fire (89030 ha), the 1979 Creek Road Fire (13235 ha), the 1985 Wheeler #2 (49649 ha), the 1985 Ferndale Fire (18941), and the 1993 Steckel Fire (11252 ha) (Fig. S3A). The two most important fires for the three southern watersheds (Harmon Canyon-Santa Clara, Arundell Barranca-Frontal Pacific Ocean, and Lower Ventura River) are the 1929 Sulpher Springs and the 1979 Creek Fire (but see FRID discussion). The Thomas Fire footprint (114,037) is shown in Fig. S3B. Fig. S3C shows fires that have occurred within the general Thomas fire area from 2018 to 2022. Recent 2025 fires in southern California are not shown. Interestingly most of the post-Thomas Fires have occurred to the north and south of our three-watershed study area (Fig. S3D).


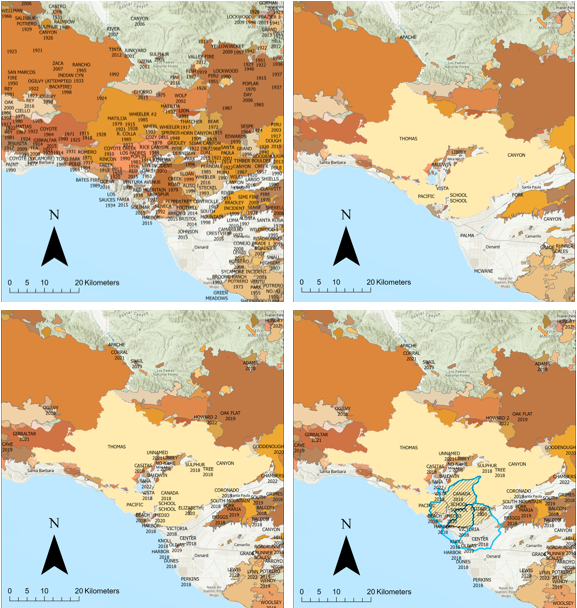


**Fig. S3.** Spatial Fire History in Thomas Fire area 1898 – 2022. Sonoma County Ca. Panel A. 1898 to 2016 Wildfires. Panel B. 2017 Thomas fire. Panel C. Fires 2018 through 2022. Panel D. Boundaries of the three southern watersheds: Harmon Canyon-Santa Clara, Arundell Barranca-Frontal Pacific Ocean, and Lower Ventura River (blue border/cross hatch).

**Fire Severity – Tubbs Fire**

Fire severity one year after the Tubbs Fire (Fig. S4) was generally low after the fire with 87% of the burned area within the Mark West watershed observed as either low (61%) or moderate (25%). High severity fire was predominant in the forested areas (99% of high severity patches), particularly conifer dominated landscapes (94% of high severity patches, see FRID discussion below).

In Mark West watershed

- Low: 3420.5 ha (8452.2 acres) 61%
- Moderate: 1419.3 ha (3507.1 acres) 25%
- High: 717.8 ha (1773.6 acres) 13%
- Increased greenness: 5.94 ha (14.7 acres) 1%

**Fire Severity – Thomas Fire**

Observed fire severity was generally low one-year post-Thomas Fire in the three southern watersheds (Fig. S5). This is likely a reflection of the post-fire vegetation response of shrubland species. That is, vegetation classified as chapparal or coastal shrublands tend to resprout after fire so one year later the relative difference in spectral patterns (color change) observed by satellites pre- and post-fire is mitigated. It is likely that fire severity immediately after the fire is much higher than fire severity one-year post-fire due to the rapid recover of shrub vegetation characteristic of coastal southern California ecosystems.

a) Harmon Canyon-Santa Clara watershed: 2330.6 ha; 5758.8 acres

- Low: 2090.8 ha (5166.3 acres) 90% of burn area
- Moderate: 163.8 ha (404.7 acres) 7% of burn area
- High: 1.5 ha (3.8 acres) < 1% of burn area
- Increased greenness: 74.4 ha (184.0 acres) 3% of burn area

b) Arundell Barranca-Frontal Pacific Ocean: 3193.1 ha; 7890.2 acres

- Low: 2759.4 ha (6818.5 acres) 86% of burn area
- Moderate: 346.3 ha (855.8 acres) 11% of burn area
- High: 2.6 ha (6.4 acres) < 1% of burn area
- Increased greenness: 84.8 ha (209.5 acres) 3% of burn area

c) Lower Ventura River: 8511.5 ha; 21031.9 acres

- Low: 7513.1 ha (18564.9 acres) 88% of burn area
- Moderate: 701.5 ha (1733.3 acres) 8% of burn area
- High: 1.4 ha (3.3 acres) < 1% of burn area
- Increased greenness: 295.6 ha (730.3 acres) 3% of burn area

Total fire severity in three watersheds burned area

- Low: 12363.3 ha (30549.71 acres) 88% of burn area
- Moderate: 1211.6 ha (2993.8 acres) 9% of burn area
- High: 5.49 ha (13.6 acres) < 1% of burn area
- Increased greenness: 454.8 ha (1123.7 acres) 3% of burn area


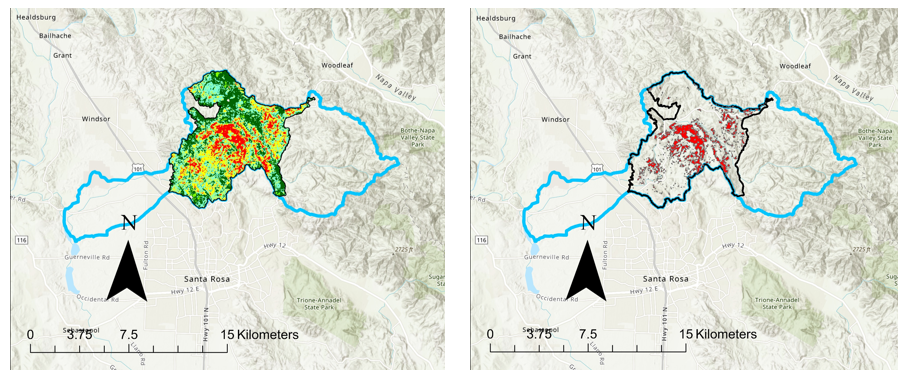


**Fig. S4**. Tubbs fire severity within the Mark West watershed. Panel A. Spatial variability in fire severity. Dark Green: Unburned to low. Teal: Low fire severity. Yellow: Moderate fire severity. Red: High fire severity. Low to high indicates increased vegetation mortality and biomass consumption. Panel B. Location of high severity patches. High severity indicates >75 percent vegetation mortality.


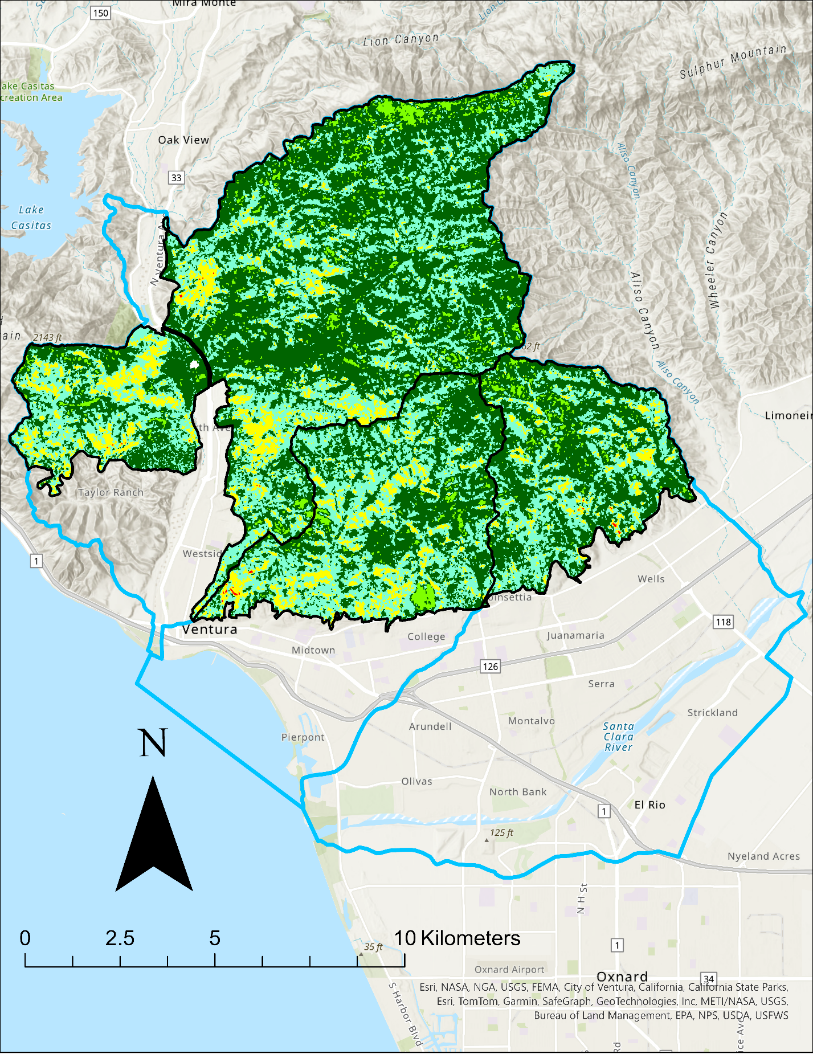


**Fig. S5.** Fire severity within the portions of the Harmon Canyon-Santa Clara, Arundell Barranca-Frontal Pacific Ocean, and Lower Ventura River watersheds burned during the 2017 Thomas Fire.

**High Severity Patch Size – Tubbs Fire**

Most of the high severity fire was in small, isolated patches less than 4 ha (10 acres) in size (Fig. S4). <4 ha (< 10 acres) = 484 polygons, 185 ha (457 acres), 4 to 40 ha (10 to 100 acres) = 22 polygons, 484.4 ha (1197 acres); 1 polygon > 40 ha (100 acres); 3 polygons >20.2 & < 40 ha (>50 & < 100 acres), 7 polygons > 10.1 & 20.2 ha (>25 acres & <50 acres), 101.2 to 404.7 ha (250 to 1000 acres) = 1 polygon, 205.6 ha patch (508 acres)

High severity fire was virtually restricted to forest vegetation types (99% of high severity burn area) with most of the forests burned at high severity classified as conifer dominated (94% of forest burned at high severity).

**High Severity Patch Size – Thomas Fire**

Fire severity one-year post-fire within the Thomas fire was focused on low and moderate severity. Very few areas were classified as high severity. Twenty-seven high severity patches were located but no high severity fire patches were larger than 0.6 ha. Total area covered by high severity equaled <4 ha (< 10 acres) = 27 polygons grouped into <4 ha (< 10 acres) patches, no polygons larger than 4 ha were observed. Total area covered by high severity patches equaled 3.9 ha (9.5 acres); <1% of area burned by Thomas Fire in the three southern watersheds.

**Fire Return Interval Departure: FRID – Tubbs Fire**

The 2016 FRID dataset provided useful information for the state of the landscape prior to the Tubbs Fire. FRID 2016 reported 408 polygons within the Tubbs Fire – Mark West burn area before the fire. For chapparal dominated landscapes burned in the Tubbs fire little change in fire return intervals was noted when comparing the reference period (pre-Euro American settlement) to the pattern of current fires. The fire regime in this vegetation type remained consistent over time burning roughly every 50 years as a stand replacing fire where the vegetation is top killed but rapidly recovers. The fire return interval departure value of -1 indicates the area is burning slightly more often than historically but the mean reference fire return interval of 55 is not significantly different than the current FRI of 54 years.

The forest vegetation patterns indicate there has been significant fire regime change in landscapes dominated by forest vegetation within the Mark West watershed. In forest ecosystems the mean reference period FRI ranged from 11 to a high of 29 years (see data by vegetation type below). While the current FRI before the Tubbs fire was 54 years. The fire return interval departure of 2 or 3 indicates that the area is burning much less frequently than it was historically. Moreover, the Fire Regime I designation for pre-Euro American fires when compared with the observed fire severity in forest dominated landscapes indicates current fires are burning at high fire intensity and result in higher fire severity than expected in the past. This is most likely due to the increased vegetation growth (grasses, small trees, and shrubs) under the canopy of forested landscapes.

**Fire Return Interval Departure: FRID 2016 – Thomas Fire Area**

The 2016 FRID dataset for the Thomas Fire area shows that the pre-Thomas fire landscape was dominated by chapparal and coastal shrublands. Very little area is covered by forest vegetation, and what is categorized as forest is predominantly evergreen oaks. Overall, the area can be classified as a landscape characterized by Fire Regime IV, that is historic fires burned as high intensity, stand replacing fires where the vegetation is top killed but responds and recovers rapidly post-fire. However, the pre-Thomas fire FRID data provides information to support the fire history conclusion reported in the literature that these southern California landscapes areas are burning more frequently than they did historically. The FRID values of -2 or -3 indicate that the Thomas Fire landscape is burning much more frequently than in the past.

**FRID – Fire Return Interval description**

The fire return interval is essentially the time between successive fires, or the frequency that an area is likely to burn. Fire frequency, along with intensity, seasonality, severity, fire type, fire size, and fire return interval help define the fire regime, that is, the pattern of fire behavior expected within a landscape.

FRID is a spatial data set that provides a comprehensive assessment of the pre-Euro-American fire regimes across California (Van de Water and Safford 2011). Essentially, FRID compares scientifically based estimates of historic fire return intervals with current patterns for every landscape in California. FRID is updated annually and maintained by the USDA Forest Service and CalFire’s FRAP program. The mean reference period refers to the Pre-Euro American settlement time-period – three or four centuries pre-settlement.
